# Distinct Brain Systems Mediate the Effects of Nociceptive Input and Self-Regulation on Pain

**DOI:** 10.1371/journal.pbio.1002036

**Published:** 2015-01-06

**Authors:** Choong-Wan Woo, Mathieu Roy, Jason T. Buhle, Tor D. Wager

**Affiliations:** 1 Department of Psychology and Neuroscience, University of Colorado, Boulder, Colorado, United States of America; 2 Institute of Cognitive Science, University of Colorado, Boulder, Colorado, United States of America; 3 Department of Psychology, Columbia University, New York, New York, United States of America; University of Oregon, United States of America

## Abstract

Cognitive self-regulation can strongly modulate pain and emotion. However, it is unclear whether self-regulation primarily influences primary nociceptive and affective processes or evaluative ones. In this study, participants engaged in self-regulation to increase or decrease pain while experiencing multiple levels of painful heat during functional magnetic resonance imaging (fMRI) imaging. Both heat intensity and self-regulation strongly influenced reported pain, but they did so via two distinct brain pathways. The effects of stimulus intensity were mediated by the neurologic pain signature (NPS), an *a priori* distributed brain network shown to predict physical pain with over 90% sensitivity and specificity across four studies. Self-regulation did not influence NPS responses; instead, its effects were mediated through functional connections between the nucleus accumbens and ventromedial prefrontal cortex. This pathway was unresponsive to noxious input, and has been broadly implicated in valuation, emotional appraisal, and functional outcomes in pain and other types of affective processes. These findings provide evidence that pain reports are associated with two dissociable functional systems: nociceptive/affective aspects mediated by the NPS, and evaluative/functional aspects mediated by a fronto-striatal system.

## Abstract 2

Two distinct parallel neural systems independently contribute to our overall experience of pain – separately modulated by noxious input and by cognitive self-regulation.

## Author Summary

Does cognitive self-regulation influence pain experience by affecting the primary representations of painful (nociceptive) stimuli in the brain? Or does it regulate reported pain via a neural pathway that is distinct from the one that mediates nociceptive pain? The present study demonstrates that nociceptive and cognitive manipulations of pain influence two distinct, separable neural systems, which operate together to construct the pain experience. The neurologic pain signature (NPS) mediates the effects of noxious input, whereas a fronto-striatal pathway connecting nucleus accumbens and ventromedial prefrontal cortex mediates the effects of cognitive self-regulation of pain. These findings help move the field beyond the “one system” view of pain as a primarily nociceptive process, and provide a foundation for new approaches to multidimensional pain assessment and treatment.

## Introduction

The ability to regulate affective experience, including negative emotion and pain, is critical for physical and mental health [1]. There is currently intense interest in the brain mechanisms that underlie self-regulatory strategies [2],[3] and the types of brain processes they influence. Current theories about the cognitive regulation of pain and emotion suggest that shifts in cognitive context act to modify primary affective processes, effectively “turning up” or “turning down” bottom-up nociceptive and affective signals in the brain. For example, cognitive reappraisal of emotional images—an important type of affective self-regulation—consistently influences functional magnetic resonance imaging (fMRI) activity in the amygdala [3]–[6] along with reports of negative emotion. These findings have typically been taken as evidence that self-regulation modulates bottom-up affective brain processes. However, fMRI activity within the amygdala is not an adequate marker for bottom-up negative affect, as it is not specific to negative affective experience [7]–[9]. Therefore, whether and how self-regulation influences primary affective representations still needs to be answered.

Pain provides a particularly developed platform for assessing the effects of self-regulation. Self-generated context and imagery can influence pain [10], and self-regulatory strategies are integral to cognitive behavioral treatments of chronic pain [11]. At the brain level, much work has been devoted to identifying ascending nociceptive systems and their central nervous system targets (e.g., anterior and dorsal-posterior insula [a/dpINS], anterior cingulate cortex [ACC], primary and secondary somatosensory cortices [S1/S2], and medial thalamus [12],[13]) and to showing that these cortical and subcortical brain areas correlate with the intensity of noxious input and pain independent of input [14]–[17]. There are few studies testing the effects of self-regulation on these systems (cf. [18],[19]), though other manipulations of cognitive context—e.g., expectations [20],[21], emotion [22]–[24], relative comparisons between pain and relief [25], and distraction induced by secondary tasks [23],[26],[27]—have been shown to influence identified pain-related targets, suggesting that various forms of cognitive regulation act on the same systems as nociceptive input.

Our goal in the present study was to test whether self-regulation influences pain via effects on primary nociceptive/affective systems or other, evaluative/functional aspects of pain dissociable from nociception. This study extends work on “top-down” modulation of pain to self-regulatory processes most commonly studied in emotion. More fundamentally, however, it was aimed at providing a stronger test of what types of brain representations are affected by “top-down” modulation, which can be applied to other treatments as well (e.g., expectancy, conditioned pain modulation, or even drug treatments).

A central problem in achieving this goal is that even though pain has relatively well-defined primary targets (e.g., compared to emotion), pain is a multidimensional experience, with sensory, cognitive, and evaluative aspects [28],[29]. The brain systems representing each aspect have not been clearly differentiated. For example, even though a few groundbreaking studies have demonstrated “top-down” modulation of spinal cord activity [30]–[32], it is unclear the degree to which these responses (a) reflect sensory versus affective aspects of pain and (b) are large enough to fully explain top-down modulation. As in the brain, multiple spinal pathways carry differential information about the location, affective qualities, and suffering components of pain (e.g., [13]). Even more fundamentally, the coarse-scale activations typically used to assess pain-related fMRI activity throughout the cerebrum are not specific to pain. Affective and sensory experiences that are clearly distinct from somatic pain also activate most or all of the identified “pain-related” regions [33]–[35], and the regions most strongly associated with pain affect (the aversiveness of pain; i.e., dorsal anterior cingulate cortex [dACC] and anterior insula [aINS]) are the most frequently activated brain areas in fMRI studies across all task types [36].

To address these issues, we employed a novel combination of two approaches. First, to test whether self-regulation and other treatments influence pain-related brain responses, brain markers sensitive and specific to pain must be used [16],[37],[38]. Multivariate patterns of fMRI activity can provide much more powerful and specific targets for process dissociation than traditional brain-mapping responses [38]–[40]. Second, to dissociate two aspects of pain (e.g., sensory versus affective, or sensory/affective versus evaluative), studies must identify experimental manipulations that independently manipulate each aspect. If manipulation A affects brain process 1 but not 2, and manipulation B affects brain process 2 but not 1, then the brain processes are said to be “separately modifiable” [41]. The separate modifiability criterion, and a weaker form called double dissociation, have been one of the main ways of dissociating mental processes in neuropsychological studies for decades [41],[42]. However, they have seldom been applied to fMRI studies.

In this study, we incorporated both approaches to provide a strong test of whether self-regulation influences primary nociceptive/affective processes, or conversely whether noxious input and self-regulation influence separately modifiable brain systems in a manner detectable with fMRI. Participants (*n* = 33) experienced thermal stimulation at six distinct temperatures during fMRI scanning (44.3–49.3°C in 1-°C increments on the left forearm) (Figure S1). These temperatures are all in a range that activates TRPV1 nociceptive ion channels [43]. On some runs, participants implemented a cognitive self-regulation strategy directed at either increasing (“regulate-up”) or decreasing (“regulate-down”) pain. The strategy is similar to reappraisal procedures commonly used to “rethink” responses to images and events [2], which also involve a mix of mental imagery and subvocalized narrative. Our intervention was designed to particularly target both sensory and affective components of pain based on effective self-regulation strategies used in prior pain studies (for the full instructions, see Materials and Methods) [10].

To test the effects of noxious stimuli and self-regulation on nociceptive/affective versus evaluative/functional aspects of pain, we examined the effects of both manipulations on two *a priori* brain systems identified in prior literature. One was the “neurologic pain signature” (NPS)—a distributed pattern of fMRI activity shown to sensitively and specifically track pain intensity induced by noxious inputs across four studies (see Results for a brief summary) [16]. This pattern includes brain regions associated with both sensory and affective aspects of pain (we did not attempt to dissociate them here), and therefore, it provides a provisional brain marker for primary nociceptive/affective pain responses. The second system was a fronto-striatal pathway connecting ventromedial prefrontal cortex (vmPFC) and nucleus accumbens (NAc, or ventral striatum), which has been shown to be important in both reappraisal studies [44]–[47] and functional and modulatory aspects of pain [48]–[51]. In humans, vmPFC activity tracks spontaneous pain when it has become chronic (and potentially dissociated from nociception) [52],[53], and vmPFC-NAc connectivity predicts the subsequent transition to chronic pain [54]. In animal models, opioid, dopamine, and NK1 pathways in the NAc are critical for behavioral modulation of pain [55]–[58], and structural reorganization in NAc and vmPFC occurs after partial nerve injury and is linked to the development of neuropathic pain [59],[60].

**Figure 1:**
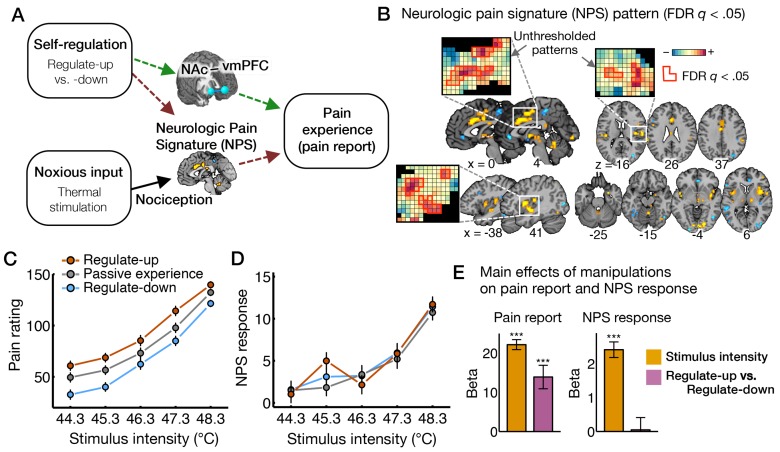
The effects of nociceptive input and cognitive self-regulation on reported pain and brain activity. (A) Hypotheses about the effects of cognitive regulation on pain-related brain processes. The effects of cognitive self-regulation on pain could be mediated by the NPS (hypothesis A, dark-brown dashed lines) or other brain systems, particularly a fronto-striatal pathway connecting vmPFC and NAc (hypothesis B, green dashed lines). (B) The NPS pattern, an *a priori* distributed pattern of fMRI signal that is sensitive and specific to physical pain [16]. Here, we present the thresholded pattern map (*q*<0.05, false discovery rate [FDR]) for display only, but all voxels within NPS were used in analyses. Some examples of unthresholded patterns are also presented in the insets; small squares indicate voxel weights, and black squares indicate empty voxels located outside of the NPS mask. (C,D) Pain ratings and NPS response as a function of stimulus intensity and regulation conditions. Because the highest level of heat intensity, 49.3°C, was not used in the self-regulation runs, we displayed results only for five levels of stimulus intensity. However all levels of heat intensity were included in analyses. NPS response values, which indicate the strength of expression of the signature pattern, are calculated by taking the dot-product of the NPS pattern weights and activation maps for each single trial. Error bars represent within-subject standard errors of the mean (SEM). The numerical data used to generate the plots can be found in Table S1. (E) The main effects of manipulations (stimulus intensity and regulate-up versus -down) on pain ratings and NPS response. Beta values (y-axis) represent regression coefficients from a multilevel generalized linear model. Error bars represent SEM. ^***^ *p*<0.001, two-tailed.

With regard to the effects of self-regulation, we hypothesized two types of effects (which are not mutually exclusive). According to “hypothesis A” (dark-brown in Figure 1A), self-regulation may reduce pattern responses in the NPS and its constituent regions, consistent with the descending modulation of nociception and/or pain affect [61]–[63]. Such effects would be consistent with effects on primary nociceptive/affective responses and imply that self-regulation influences the same systems targeted by ascending nociceptive input. Alternatively, according to “hypothesis B” (green in Figure 1A), self-regulation may affect other brain systems linked to pain, such as the vmPFC-NAc system.

We found that both nociceptive input and self-regulation strongly influenced pain. However, the NPS mediated only the effects of nociceptive input. The self-regulation effects on pain were mediated by a pathway connecting the NAc and vmPFC, which was unresponsive to the intensity of nociceptive input. The NAc-vmPFC system (a) responds to self-regulation in both “increase” and “decrease” directions; (b) predicts pain on a trial-by-trial basis; and (c) is independent of the NPS and nociceptive input. Because it predicts pain as defined by the clinical and experimental “gold standard” of self-report [64],[65] and because of the large literature on pain behavior in animals referenced above, activity in the NAc-vmPFC system cannot be dismissed simply as “response bias.” Rather, the findings demonstrate the separate modifiability of two pain-related systems by “bottom-up” and “top-down” input, and suggest that the NAc-vmPFC system plays a role in functional/evaluative aspects of pain that are dissociable from nociception. Identifying these pathways helps expand the view of pain beyond medial/lateral “pain-processing” systems identified primarily using manipulations of nociception, and opens the way for quantitative multidimensional assessments of pain neurophysiology.

## Results

### Characteristics of the Neurologic Pain Signature in Prior Studies

The NPS is a distributed pattern of fMRI activity recently shown to be highly sensitive and specific to pain as manipulated by nociceptive input (Figure 1B) [16]. It is defined by meso-scale patterns within both medial (e.g., “affective”; anterior cingulate cortex) and lateral (e.g., “sensory”; somatosensory cortices) “pain systems” [66] that are consistent across individuals. Across four fMRI studies, the strength of the NPS response discriminated somatic pain from non-painful warmth, pain anticipation, distressing images related to social rejection, and pain recall in 90%–100% of individual participants tested [16]. Importantly, the NPS is not likely to be simply a measure of noxious input: It tracked subjective pain more closely than the noxious stimulus itself (both in intensity and time-course), responds more strongly when pain is more intense at a fixed noxious stimulus intensity, and was substantially reduced by the opiate analgesic remifentanil. Though “pain intensity” and “pain affect” are typically highly correlated (*r*>0.9 [67]) and were not dissociated in these studies, the NPS includes regions most closely linked to sensory/discriminative (e.g., S2/dpINS [68],[69]) and affective (e.g., dACC and aINS [70],[71]) aspects of pain. Thus, these findings establish the NPS as a brain marker for a component of pain likely related to a combination of nociceptive and affective aspects.

### Behavioral Results

Both heat intensity and cognitive self-regulation substantially impacted pain reports. As expected, pain reports were higher with increasing heat intensity (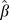 = 22.10, *t*_32_ = 17.60, *p*<0.0001). In addition, pain increased in the regulate-up (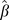 = 14.42, *t*_32_ = 3.71, *p*<0.001) and decreased in the regulate-down (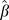 = −13.39, *t*_32_ = −3.82, *p*<0.001) compared to the passive experience condition, and pain was higher in the regulate-up than regulate-down condition (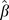 = 14.02, *t*_32_ = 4.75, *p*<0.0001) (Figure 1C and 1E; Table S1). There was no interaction effect on pain between heat intensity and self-regulation instructions (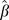 = −0.66, *t*_32_ = −1.03, *p* = 0.31). Further, the significant effects of self-regulation on pain were retained when considering only data in the clearly noxious range, above 47°C (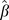 = 12.62, *t*_32_ = 4.13, *p*<0.001) and above 48°C (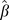 = 10.97, *t*_32_ = 3.68, *p*<0.001), which were judged to be painful on 82% and 95% of trials for all participants, respectively. We also tested the effects of heat intensity and self-regulation (regulate-up versus -down) on the pain versus no-pain decision using multilevel logistic regression. Both heat intensity and self-regulation significantly influenced the percentage of pain decision (Figure S2; 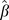 = 1.70, *t*_31_ = 12.98, *p*<0.001, and 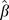 = 1.47, *t*_31_ = 2.77, *p*<0.01) with no interaction effects between stimulus intensity and self-regulation (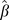 = −0.28, *t*_31_ = −1.56, *p* = 0.13). These additive effects of self-regulation and stimulus intensity on pain provide a clue that the effects of self-regulation might not interact with nociceptive processes.

### Neurologic Pain Signature Responses Mediate Pain Induced by Peripheral Stimulation but Not Self-Regulation

**Figure 2:**
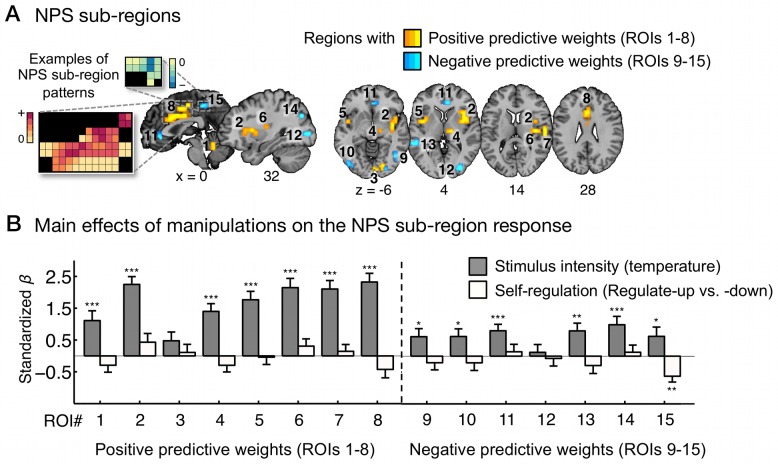
The effects of stimulus intensity and self-regulation on separate sub-regions within the neurologic pain signature. (A) NPS sub-regions: ROIs 1–8 are regions with positive NPS weights, and ROIs 9–15 are regions with negative NPS weights. We obtained these regions from the FDR (*q*<0.05) thresholded NPS map (see Figure 1B) smoothed with a 0.5 mm Gaussian kernel. Some examples of weight patterns within the NPS sub-regions are presented in the insets; small squares indicate voxel weights, and black squares indicate empty voxels located outside of the ROIs. (B) The main effects of stimulus intensity and self-regulation on the NPS response within sub-regions. y-Axis represents standardized regression coefficients from multilevel generalized linear models with stimulus intensity and self-regulation (regulate-up versus -down) as predictors and NPS response as dependent variables. Error bars represent standard errors of the mean (SEM). The data used to generate the plots can be found in Table S2. ***p<0.001; **p<0.01; *p<0.05, two-tailed.

To test whether self-regulation affects pain reports by changing primary nociceptive and affective brain processes (hypothesis A), we estimated the linear increase in NPS response as a function of both stimulus intensity and regulate-up versus regulate-down instructions (Figure 1D and 1E; Table S1). NPS response increased substantially with stimulus intensity (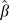 = 2.41, *t*_32_ = 10.53, *p*<0.0001), but showed no effect of self-regulation (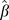 = 0.05, *t*_32_ = 0.13, *p* = 0.90) and no interaction effect by stimulus intensity and self-regulation (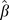 = −0.03, *t*_32_ = −0.20, *p* = 0.84). This pattern of findings was the same in the clearly noxious range, above 47°C and 48°C: There was a strong effect of stimulus intensity on NPS responses (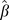 = 5.14, *t*_32_ = 7.95, *p*<0.0001 for trials above 47°C, and 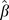 = 1.81, *t*_32_ = 2.41, *p*<0.05 for trials above 48°C), but no effect of self-regulation on NPS responses (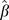 = 0.07, *t*_32_ = 0.12, *p* = 0.91 for trials above 47°C, and 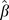 = −0.32, *t*_32_ = −0.51, *p* = 0.61 for trials above 48°C). In addition, none of the NPS sub-regions showed significant effects of self-regulation on the local NPS pattern response except for the precuneus, which showed increasing deactivation with stimulus intensity but increasing activation with regulate-up versus -down (Figure 2; Table S2).

**Figure 3:**
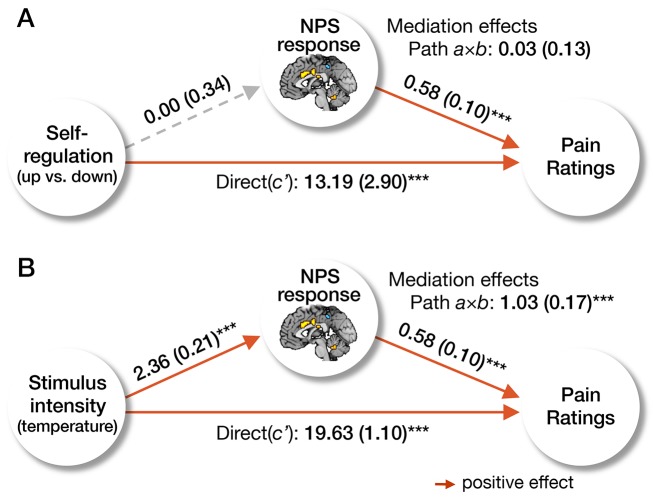
Mediation of the neurologic pain signature. We present the results of the multilevel mediation analyses with the NPS response as a mediator. In (A), cognitive self-regulation (regulate-up versus -down instructions) was entered as a predictor, and in (B), stimulus intensity (i.e., temperature) was entered as a predictor. In both models, pain report was an outcome, and the other manipulation (e.g., stimulus intensity for model A, and self-regulation for model B) was entered as a covariate. The results showed that the NPS response mediated the effects of stimulus intensity on pain, but did not mediate the effects of cognitive regulation on pain. The paths (path *a*, *b*, and *c′*) and mediation effects (path *a*×*b*) are labeled with path coefficients, and their standard errors are shown in parenthesis. The gray dashed line indicates a non-significant path. ***p<0.001, two-tailed.

In addition, we used multilevel mediation [20] to test whether the NPS response mediated the effects of both self-regulation and stimulus intensity on pain report. Mediation analysis tested the joint effects of self-regulation on NPS response (path *a*) and NPS response on pain (path *b*), as well as the total (path *c*) and direct (non-mediated) effect of self-regulation on pain (path *c′*). Regulate-up versus -down instructions and NPS response magnitude were both associated with increased pain ratings (Figure 3A, paths *b* and *c*), but self-regulation instructions did not impact the NPS response (path *a*), and therefore the mediation effect (path *a*×*b*) was not significant. In contrast, the NPS response mediated the effect of heat intensity on pain ratings (Figure 3B). Therefore, hypothesis A was not supported by the results, indicating that the pain-modulatory effects of self-regulation are not primarily mediated by changes in primary nociceptive and affective processes.

### Voxel-Wise Brain Maps Show Separate Targets for Noxious Stimulus Intensity and Self-Regulation

Standard voxel-wise mapping supported the conclusion that noxious input, but not self-regulation, impacted targets of ascending nociceptive pathways. Increasing stimulus intensity was associated with increased activity in a number of regions associated with nociceptive processing and pain construction [12],[15],[17], including right (contralateral) dpINS, bilateral S2, bilateral aINS, ventrolateral and medial thalamus (vl/mThal), and dACC (Figure S3; Table S3; all voxel-wise results reported are significant at cluster-level *p*<0.05, family-wise error rate [FWER] corrected).

**Figure 4:**
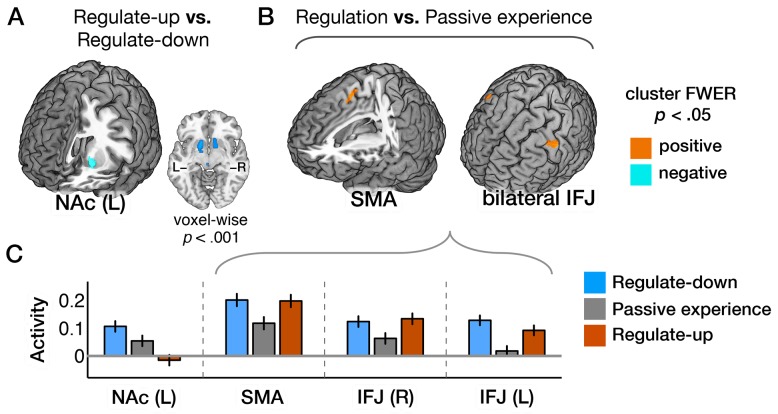
Brain activity induced by self-regulation. (A) Activity in left NAc was associated with regulate-up versus regulate-down instructions (at *p*<0.05, FWER corrected based on cluster extent, with a primary threshold of *p*<0.0005, *k*>84). Bilateral activations were found at a lower threshold (voxel-wise *p*<0.001). (B) Activity in the supplementary motor area (SMA) and bilateral inferior frontal junction (IFJ) were associated with regulation versus passive experience instructions. (C) Bar plots of the averaged activity (y-axis) within the corresponding brain regions for conditions (x-axis). Error bars represent within-subject standard errors of the mean (SEM). The data used to generate the plots can be found in Table S4.

In contrast, cognitive self-regulation (regulate-up versus -down contrast) yielded a very different pattern of results. The correlation between the map of stimulus intensity effects and the self-regulation effects was *r* = 0.02 across all voxels, and *r* = −0.13 across voxels in the Neurosynth meta-analytic map for “pain” [36]. Rather than affecting the main targets of nociceptive afferents, regulate-down enhanced, and regulate-up suppressed activity in the left NAc relative to passive experience (Figure 4A and 4C; Table S4). The effects in NAc were bilateral and extended into ventral putamen at a slightly lower threshold (voxel-wise *p*<0.001, uncorrected). The NAc was unresponsive to stimulus intensity (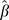 = −0.002, *t*_32_ = −0.33, *p* = 0.76), suggesting that it does not represent primary nociceptive information. A portion of the caudate nucleus also showed the same pattern of the self-regulation effects (Figure S4). Conversely, sensorimotor cortex showed greater activity in regulate-up versus -down conditions (Figure S4).

We note that we did find a significant increase in dpINS/S2 activity in regulate-up versus passive experience (Figure S5A), which is one of main targets of ascending nociceptive afferents [12],[15]. However, there was no significant increase in dpINS/S2 activity in regulate-up versus -down, and no other ascending nociceptive targets showed increased activity in regulate-up versus passive experience. In addition, the NPS response was not different for regulate-up versus passive experience, either for whole-brain pattern responses (*t*_32_ = 1.41, *p* = 0.16) or the local NPS pattern response within the dpINS/S2 (*t*_32_ = 1.84, *p* = 0.07). Thus, the self-regulation effects on some voxels within the dpINS/S2 do not constitute compelling evidence for the modulation of nociceptive responses.

Finally, as in previous studies [2],[3],[6],[72], several prefrontal regions showed increasing activity in both regulate-up and regulate-down conditions, presumably reflecting demand on context-generation systems. These included the supplementary motor area (SMA) and bilateral inferior frontal junction (IFJ) (Figure 4B and 4C). Conversely, activity in superior parietal lobe decreased in both regulate-up and regulate-down conditions relative to passive experience (Figure S4B and S4C).

### A Nucleus Accumbens-Ventromedial Prefrontal Pathway Mediates Effects of Self-Regulation on Pain

If self-regulation does not impact primary nociceptive and affective pain circuits, it may impact pain through other brain systems, particularly the NAc-vmPFC system (hypothesis B). We tested whether the NAc-vmPFC pathway mediated self-regulation effects on pain with a three-path multilevel mediation analysis using *a priori* regions-of-interest (ROIs) in the NAc (a 6-mm sphere around the center, MNI: 10, 12, −8) and vmPFC (a 6-mm sphere around the center, MNI: 2, 52, −2) based on Baliki and colleagues [53]—as they provide coordinates for ROIs tested across multiple patient cohorts. The vmPFC ROI is located in the boundary between Vogt's peri-genual anterior cingulate [73] and the rostral medial prefrontal cortex.

**Figure 5:**
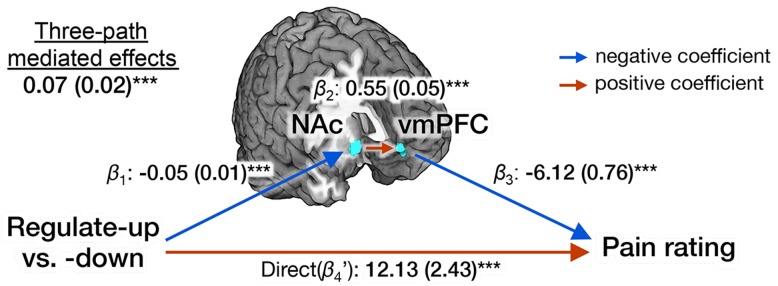
Multilevel three-path mediation analysis with two *a priori* regions-of-interest. *A priori* ROIs include the NAc (MNI: 10, 12, −8) and the vmPFC (MNI: 2, 52, −2) from Baliki and colleagues [53]. Stimulus intensity and the NPS response were included as covariates. The paths are labeled with path coefficients, and standard errors are shown in parentheses (for more details about three-path mediation analyses, see Materials and Methods). ^***^ *p*<0.001, two-tailed.

The NAc-vmPFC pathway was a significant, positive mediator of the relationship between cognitive self-regulation and pain ratings; each link of the pathway from self-regulation to pain report was significant (self-regulation→NAc→vmPFC→pain report), as well as the overall mediation effects (see Figure 5 for statistics). As Figure 5 shows, NAc activity was highest in the regulate-down condition, and NAc activity was positively associated with vmPFC activity. VMPFC activity, in turn, predicted reduced pain. Reversing the direction of the mediation, i.e., self-regulation→vmPFC→NAc→pain report, yielded non-significant results, in keeping with our voxel-wise findings and previous work suggesting that the NAc is more directly affected by self-regulation than is the vmPFC [3],[6]. Importantly, the mediation of the NAc-vmPFC pathway was significant controlling for the stimulus intensity and NPS response. To compare the effect sizes of two separate, independent contributions to pain (one through the NPS, and the other through the NAc-vmPFC pathway), we conducted a multilevel multiple regression. Each effect on pain (NPS and vmPFC controlling for NAc) was highly significant when controlling for the other, but the NPS effects on pain ratings (standardized 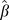 = 15.67, *t*_32_ = 9.65, *p*<0.0001) were approximately three times larger than the effects of the vmPFC (standardized 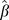 = −5.54, *t*_32_ = −5.29, *p*<0.0001). There was no interaction effect between the NPS and vmPFC on pain ratings.

**Figure 6:**
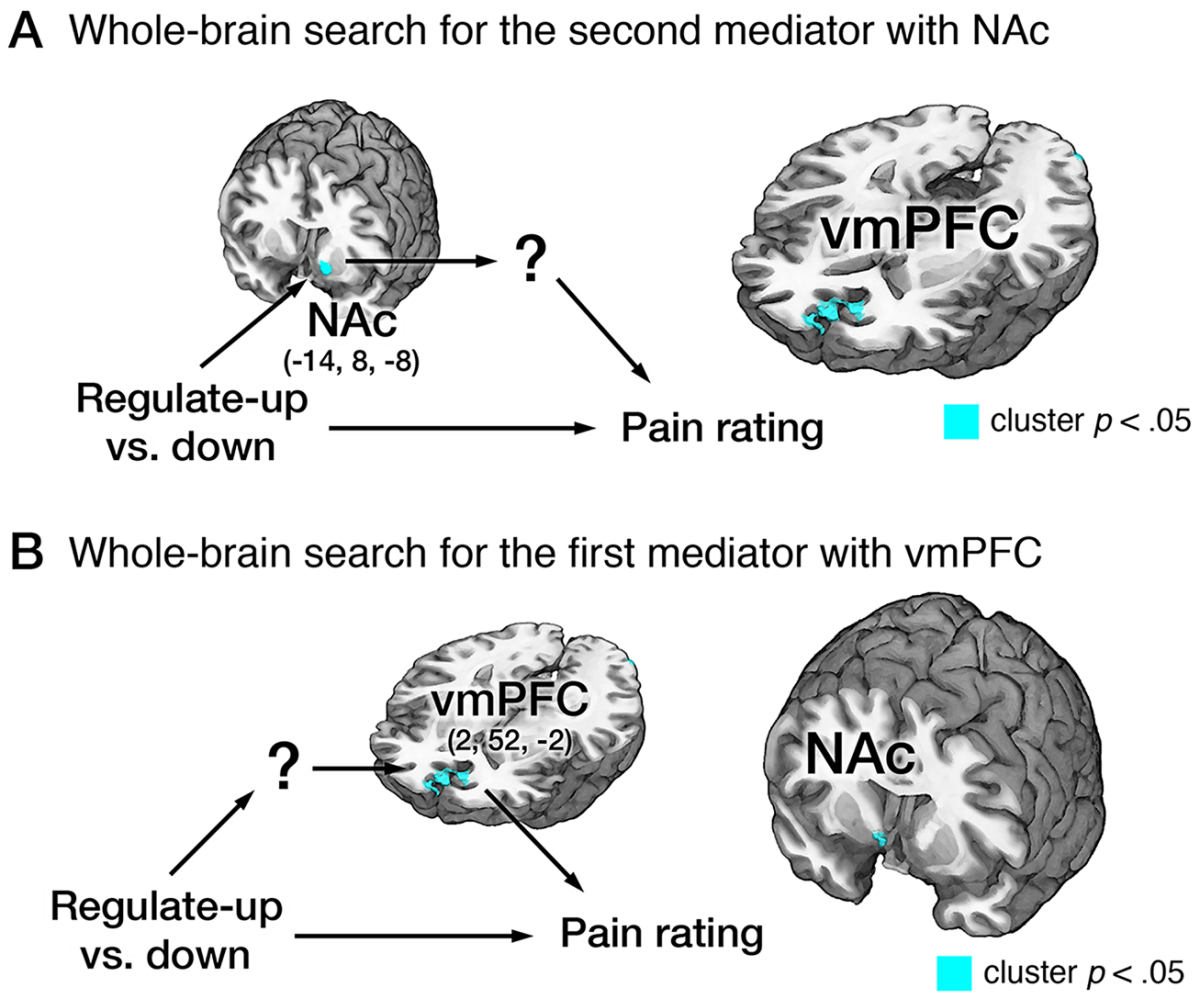
Whole-brain three-path mediation analysis results. (A) Whole-brain three-path mediation analysis with the NAc (MNI: −14, 8, −8), which is from the GLM results shown in Figure 4A, as the first mediator. VMPFC (MNI: 2, 52, −2) was the only significant second brain mediator from the whole-brain search (*p*<0.05, FWER corrected based on cluster extent, with a primary threshold of *p*<0.001). (B) Whole-brain three-path mediation analysis with vmPFC (MNI: 2, 52, −2), which is from the results shown in Figure 6A. Right NAc (MNI: 8, 8, −6) was the only significant first brain mediator from the whole-brain search. For more details of path coefficients, see Table S5. ^***^ *p*<0.001, two-tailed.

Additional whole-brain searches using multilevel three-path mediation analysis confirmed that the NAc-vmPFC pathway was the strongest mediator of self-regulation in this study, and that important other brain regions connecting between self-regulation and vmPFC or between the NAc and pain ratings were not missed due to our *a priori* focus on the NAc-vmPFC pathway. In order to use the three-path mediation framework for the whole-brain search, three variables (self-regulation, pain ratings, and either NAc or vmPFC activity) should be specified *a priori*, and a voxel-wise search was conducted for the first or second brain mediators (Figure 6). Therefore, these analyses asked the following specific questions: Given the NAc involvement, what other brain regions than the vmPFC mediate the relationship between the NAc and pain ratings? Given the vmPFC involvement, what other regions than the NAc mediate the relationship between self-regulation and the vmPFC?

We first identified brain mediators of the relationship between the regulation-NAc connection and pain reports. VMPFC was the only significant brain mediator that survived multiple-comparison correction (Figure 6A; Table S5). We also searched for brain mediators of the relationship between regulation and the vmPFC-pain reports connection. NAc was the only significant mediator (Figure 6B; Table S5).

We also conducted exploratory analyses with a two-path mediation framework, in which we identified voxels mediating the regulate-up versus -down effects on pain ratings. Again, results confirmed that the NAc-vmPFC pathway is important for the relationship between self-regulation and pain: regulate-up versus -down was negatively associated with NAc activity in path *a*, and increased vmPFC activity predicted reduced pain in path *b* (Figure S6B). Importantly, there was little overlap between regions responsive to self-regulation (path *a*) and those predictive of pain reports (path *b*) (Figure S6B), suggesting the importance of the NAc-vmPFC pathway in connecting path *a* and *b*.

## Discussion

We found strong evidence that pain is influenced by both noxious input and cognitive self-regulation, but that they are mediated by two distinct brain systems. The effects of noxious stimulus intensity were mediated by the NPS, an *a priori* pattern shown to be diagnostic of physical pain (as opposed to other salient, affective conditions). Conversely, the effects of self-regulation were mediated by the NAc-vmPFC pathway previously shown to be important for emotion/pain regulation, and valuation in a range of contexts [6],[53],[74]–[77], but which did not respond to changes in noxious stimulus intensity.

Pain reports have served as the gold standard measure of pain in clinical settings and experimental studies [64],[65],[78]. By this standard, pain is influenced by both self-regulation and noxious input. However, this study shows that these effects on pain are mediated by two distinct, separable neural systems. The identification of these separately modifiable pathways provides a step towards a quantitative assessment of multiple neurophysiological components of pain, and ultimately the deconstruction of nociceptive and extra-nociceptive contributions to pain reports. Pain has been widely thought to arise from interactions among sensory, affective, and evaluative processes [28],[79]. The different systems involved in these multiple components have been difficult to identify, and both lateral and medial systems thought to mediate “sensory” versus “affective” aspects of pain (e.g., S2/dpINS and dACC/aINS, respectively) are strongly responsive to nociceptive input [13],[71]. Our results suggest that the NPS (including lateral somatosensory and medial limbic regions) encodes brain activity that is closely tied to primary nociceptive and affective pain processing, whereas the NAc-vmPFC pathway and related networks encode information about the evaluative or functional aspects of pain in context. Both are potentially important for the overall experience of pain, with different manipulations affecting each.

One alternative interpretation of these results is that cognitive self-regulation does not impact pain, but rather induces a sort of “decision bias.” Whether the effects of the NAc-vmPFC system on pain are merely momentary biases or are functionally significant (as they are in many decision-making studies [75],[80],[81]), dissociating them from nociceptive aspects of pain is important in both experimental and clinical contexts. However, we believe that it is premature to dismiss activity in this system as “bias,” as the weight of extant evidence argues for a more nuanced view. The basal ganglia, including the NAc, is involved in processing multiple aspects of pain [49]. The NAc, in particular, has the potential to regulate descending pain modulatory pathways [48],[57]. In addition, mesolimbic dopamine projections from the ventral tegmental area (VTA) to the NAc are important in the analgesic effects induced by placebo and stress [55],[82], pain relief [58],[83], and the changes in motivated behavior caused by chronic pain [84]. The vmPFC, rather than regions included in the NPS, correlates with spontaneous, clinical pain [52], and encodes the learned, behavioral avoidance value associated with painful stimulation [85],[86]. The morphology and function of NAc and vmPFC cells changes with the chronification of pain after nerve injury [59],[60],[84] in a way that predicts and causally affects depression-like behavior [84], and the exact NAc-vmPFC pathway we tested was recently shown to predict the transition from acute to chronic back pain [53],[54], demonstrating a functional role in pain chronification. Further, both regions have been shown to be important predictors of changes in appetitive and self-regulatory behaviors in the real world [45],[80],[81], and nearby subgenual cingulate has become an important target for treatment of depression [87]. Thus, our NAc-vmPFC pathway findings may reflect evaluative processes that play an important role in the construction of pain experience and in shaping long-term motivated behaviors and outcomes. Identifying the precise functional consequences—from momentary “biases” in decision-making to long-term changes in behavior—is beyond the scope of this paper, but is an important long-term direction for the field.

Importantly, our results do not imply that all types of cognitive pain regulation influence pain in the same way. An enduring theme in self-regulation has been that not all forms of cognitive modulation are equal, and different forms of psychological modulation can influence pain and emotion via distinct systems [88]. While self-generated thoughts appeared to be difficult to influence pain and emotion in deep ways, some forms of cognitive regulation, e.g., distraction and placebo manipulations, may regulate pain at very early stages of processing in the spinal cord in some cases [30],[31]. Even in the spinal cord, however, the presence of multiple ascending pathways with different brain targets [12] and complex modulatory circuitry [89] makes it difficult to identify spinal responses simply with nociception. Understanding and comparing the effects of multiple behavioral and pharmacological treatments—self-regulation, distraction, placebo, conditioning, and opioid and non-opioid drugs—on both cerebral and spinal responses is a critical long-term agenda for the field.

This study makes a useful contribution in this regard, by laying out a logic by which multiple independent manipulations can be used to assess the modularity of brain activity [41]. Here, this logic is coupled with the use of (a) multivariate patterns that have defined diagnostic properties across studies and greater sensitivity and specificity to pain than traditional fMRI maps, and (b) mediation analyses that can link experimental effects on brain activity with trial-by-trial variation in pain. Together, these approaches provide robust ways of identifying the fMRI signal patterns that are strongly linked to primary outcomes like pain reports and other behaviors.

This study also has limitations that should be addressed in future studies. First, we did not attempt to separately assess pain intensity and unpleasantness, though these can be dissociated with specific experimental manipulations [23],[70]. We measured overall pain because its intensity and unpleasantness aspects are typically highly correlated in healthy individuals in behavioral (e.g., *r* = 0.98 [67]) and psychophysiological assessments [90]. In addition, our manipulation was designed to regulate both sensory and affective aspects of pain. In future studies, however, looking at the separate effects of intensity and unpleasantness may be informative. Second, this study does not include autonomic measures that could provide more information on the effects of self-regulation on multiple outcomes (e.g., [72],[91]). Third, we did not have a pre-scanning training session for the self-regulation strategy, and therefore the effects of short- or long-term self-regulation practice have yet to be examined.

Overall, our findings provide a foundation for a new approach to multidimensional pain assessment and a new window into the different ways that psychological and pharmacological interventions may work to relieve pain. In combination with recent findings on the functional role of the NAc-vmPFC pathway in chronic pain [51]–[53], the current findings may have implications for understanding the neurophysiological underpinnings of clinical pain. For example, some forms of clinical pain may be caused by enhanced aversive valuation of pain, rather than resulting from dysregulation of nociceptive brain processes (e.g., [92],[93]). Our findings make it possible to measure the separate contribution of a nociceptive component and an evaluative component of pain at the brain level. This provides a new basis for characterizing healthy and dysregulated individuals in terms of multiple pain-related systems. In addition, treatments that have different impacts on the two systems we identified may have different long-term consequences for the persistence and quality of pain and other affective states.

More broadly, our findings bear on the question of what is regulated when self-regulation strategies are employed to mitigate pain and negative emotions, as in various forms of psychotherapy [11],[94],[95]. Self-regulatory strategies may not operate primarily by reducing primary affective responses. Rather, emotion and pain likely arise from interactions among multiple processes [28],[96], some more closely tied to sensory events, and others linked to evaluative and motivational processes that are also central to emotional experience. Our findings suggest that the latter class of processes, rather than the former, may be most strongly impacted by self-regulation.

## Materials and Methods

### Participants

Thirty-three healthy, right-handed participants completed the study (*M*_age_ = 27.9±9.0 years, 22 females). The sample consisted of 39% white, 33% Asian, 12% Hispanic, and 15% African American participants. All participants provided informed consent. The study was approved by the Columbia University Institutional Review Board (Protocol number AAAE3743). Preliminary eligibility was assessed with a general health questionnaire, a pain safety screening form, and an fMRI safety screening form. Participants reported no history of psychiatric, neurological, or pain disorders.

### Thermal Stimulation

Thermal stimulation was delivered to the volar surface of the left inner forearm applied using a TSA-II Neurosensory Analyzer (Medoc Ltd.) with a 16-mm Peltier thermode end-plate. The stimulation was delivered on two spots located on the middle forearm that alternated between runs. Each stimulus lasted 12.5 seconds, with 3-second ramp-up and 2-second ramp-down periods and 7.5 seconds at target temperature. Six levels of temperature were administered to the participants (level 1: 44.3°C; level 2: 45.3°C; level 3: 46.3°C; level 4: 47.3°C; level 5: 48.3°C; level 6: 49.3°C).

### fMRI Task Design

fMRI images were acquired during nine functional runs. Runs 1, 2, 4, 5, 6, 8, and 9 were “passive experience” runs, where participants passively experienced and rated the heat stimuli. Among those, run 1, 2, 4, 8, and 9 comprised 11 stimulations from level 1 (44.3°C) to 5 (48.3°C), for a total of 55 stimulations over the 5 runs (11 times each temperature). Transitional frequencies between temperature levels were counterbalanced over the 55 stimulations so that each temperature level was preceded twice by each of the five temperatures, and each of the five “passive experience” runs started with a different temperature. Runs 1, 4, and 9 began with temperature level 1, 3, or 5; Runs 2 and 8 always started with levels 4 and 2, respectively. Different presentation orders were generated for each participant. During run 5 and 6, the temperatures presented in runs 4 and 8 were all increased by one degree, so that the five levels of temperature presented in these runs spanned from level 2 (45.3°C) to level 6 (49.3°C). These runs with increased temperature were designed to examine the effects of different contexts of sensory inputs. However, given that the context effects are not the current focus, we did not focus on the effects of context manipulations. After each trial, participants judged whether the stimulus was painful or not, followed by a judgment of pain or warmth intensity on a 100-point visual analog scale. See Figure S1 for the design of each trial.

Run 3 and 7 were “regulation” runs. Participants were asked to cognitively “increase” (regulate-up) or “decrease” (regulate-down) pain intensity. These two “regulation” runs comprised ten randomly presented stimulations (twice each of the first 5 levels [i.e., from 44.3°C to 48.3°C]; and same order for regulate-up and -down conditions). Because the two “regulation” runs did not have the highest level of heat intensity, 49.3°C, we displayed results only for five levels of stimulus intensity in Figure 1. However all levels of heat intensity were included in analyses. Instructions for regulate-up and down are as follows.

#### Instructions for regulate-up

“During this scan, we are going to ask you to try to imagine as hard as you can that the thermal stimulations are more painful than they are. Try to focus on how unpleasant the pain is, for instance, how strongly you would like to remove your arm from it. Pay attention to the burning, stinging and shooting sensations. You can use your mind to turn up the dial of the pain, much like turning up the volume dial on a stereo. As you feel the pain rise in intensity, imagine it rising faster and faster and going higher and higher. Picture your skin being held up against a glowing hot metal or fire. Think of how disturbing it is to be burned, and visualize your skin sizzling, melting and bubbling as a result of the intense heat.”

#### Instructions for regulate-down

“During this scan, we are going to ask you to try to imagine as hard as you can that the thermal stimulations are less painful than they are. Focus on the part of the sensation that is pleasantly warm, like a blanket on a cold day. You can use your mind to turn down the dial of your pain sensation, much like turning down the volume dial on a stereo. As you feel the stimulation rise, let it numb your arm, so any pain you feel simply fades away. Imagine your skin is very cool, from being outside, and think of how good the stimulation feels as it warms you up.”

These instructions were similar to reappraisal procedures commonly used in cognitive regulation or emotion [4]. However, our procedures differ somewhat from standard reappraisal procedures because (a) the narrative component inherent in regulation of complex images is less applicable in pain, and (b) affective imagery is particularly suited to modulating pain [10]. Our procedures also differ from many distraction tasks because our instructions do not manipulate attention itself, but the meaning of painful sensation. The order of the two regulation runs (regulate-up and regulate-down) was counterbalanced across subjects.

### fMRI Acquisition and Preprocessing

Whole-brain fMRI data were acquired on a 3T Philips Achieva TX scanner at Columbia University's Program for Imaging in Cognitive Science (PICS). Structural images were acquired using high-resolution T1 spoiled gradient recall images (SPGR) for anatomical localization and warping to a standard space. Functional EPI images were acquired with TR = 2,000 ms, TE = 20 ms, field of view = 224 mm, 64×64 matrix, 3×3×3 mm^3^ voxels, 42 interleaved slices, parallel imaging, SENSE factor 1.5. Stimulus presentation and behavioral data acquisition were controlled using E-Prime software (PST Inc.).

Structural T1-weighted images were coregistered to the mean functional image for each subject using the iterative mutual information-based algorithm implemented in SPM8 and manual adjustment of the starting point until the coregistration was satisfactory. Structural images were normalized to MNI space using SPM8. Prior to preprocessing of functional images, we removed the first four volumes to allow for image intensity stabilization. We also identified image-wise outliers by computing both the mean and the standard deviation (across voxels) of intensity values for each image for all slices to remove intermittent gradient and severe motion-related artifacts that are present to some degree in all fMRI data. To identify outliers, Mahalanobis distances for the matrix of slice-wise mean and standard deviation values (concatenated)×functional volumes (time) were computed, and any values with a significant *χ^2^* value (corrected for multiple comparisons based on the more stringent of either false discovery rate or Bonferroni methods) were considered outliers (less than 1% of images were outliers). The output of this procedure was later used as nuisance covariates in the first level models.

Then, functional images were corrected for differences in the acquisition timing of each slice and were motion (realignment) corrected using SPM8. The functional images were warped to SPM's normative atlas using warping parameters estimated from coregistered, high resolution structural images, interpolated to 2×2×2 mm^3^ voxels, and smoothed with an 8 mm FWHM Gaussian kernel. This smoothing level improves inter-subject functional alignment, while retaining sensitivity to mesoscopic activity patterns that are consistent across individuals [97].

### Behavioral Analysis

We analyzed the behavioral data using a multilevel generalized linear model analysis [98], implemented with custom code written in MatLab (http://wagerlab.colorado.edu/tools). The outcome variable was pain reports (linear regression) or pain decision (pain versus no-pain; logistic regression) for each trial. Within subject predictors at the first level of the model included cognitive self-regulation conditions (regulate-up, passive experience, and regulate-down were coded as 1, 0, and −1), stimulus intensity (i.e., temperature), and their interaction. A between-subject covariate included the order of regulate-up and regulate-down runs (regulate-up first versus regulate-down first were coded as 1 and −1, respectively). For the logistic regression with pain decision as an outcome variable, one participant who always chose “pain” was excluded from the analysis.

### fMRI Analysis

#### First-level analysis and robust regression

First-level general linear model (GLM) analyses were conducted in SPM8. The nine runs were concatenated for each subject. Boxcar regressors, convolved with the canonical hemodynamic response function, were constructed to model periods for the 12.5-second thermal stimulation and 11-second rating periods. We included three regressors that are parametric modulation of cognitive regulation, stimulus intensity, and their interaction, analogous to the behavioral analysis.

The fixation cross epoch was used as an implicit baseline. A high-pass filter of 180 seconds, which is well suited for longer duration pain, was applied. Other regressors of non-interest (nuisance variables) included (a) “dummy” regressors coding for each run (intercept for each run); (b) linear drift across time within each run; (c) the six estimated head movement parameters (x, y, z, roll, pitch, and yaw), their mean-centered squares, their derivatives, and squared derivative for each run (total 24 columns); (d) indicator vectors for outlier time points identified based on their multivariate distance from the other images in the sample (see above); (e) indicator vectors for the first two images in each run; (f) signals from white matter and ventricle.

Second-level analyses (group) were conducted using robust regression [99] with cognitive regulation strength (mean difference between pain ratings for regulate-up versus regulate down for each temperature level) or pain sensitivity (mean pain rating across passive experience trials) as second-level covariates for regressors for cognitive regulation and stimulus intensity, respectively. All results were thresholded at *p*<0.05, FWER corrected based on cluster extent with primary threshold of *p*<0.001, *p*<0.0005, or *p*<0.00001, two-tailed. The cluster extents for FWER correction were estimated based on Monte Carlo simulation (10,000 iterations) with 3dClustSim of AFNI (http://afni.nimh.nih.gov/) using the estimated intrinsic smoothness [100] using 3dFWHMx of AFNI. For the purpose of display, we pruned the results using two additional higher levels of voxel-wise threshold.

#### Single trial analysis

For mediation and pattern expression analyses, we employed the single trial, or “single-epoch,” design and analysis approach. There have been several papers demonstrating that single trial analyses are reliable and offer increased sensitivity, especially in modeling responses to pain [101]. In this study, quantification of single-trial response magnitudes was done by constructing a GLM design matrix with separate regressors for each trial, as in the “beta series” approach of Rissman and colleagues [102]. Similar to the parametric modulation model, boxcar regressors, convolved with the canonical hemodynamic response function, were constructed to model periods for the 12.5-second thermal stimulation and 11-second rating periods. Then, we included a trial-specific regressor for each trial, as well as nuisance covariates that are identical to above.

One important consideration in the single trial analysis is that trial estimates could be strongly affected by acquisition artifacts that occur during that trial (e.g., sudden motion, scanner pulse artifacts, etc.). For this reason, trial-by-trial variance inflation factors (VIFs, a measure of design-induced uncertainty due in this case to collinearity with nuisance regressors) were calculated, and any trials with VIFs that exceeded 2.5 were excluded from the following analyses. The average number of excluded trials was 9.55 (SD = 4.13) per subject. The single-trial beta images were used in mediation analyses (see below) and ROI analyses.

#### Pattern expression analysis

In order to calculate the strength of expression of the NPS pattern (i.e., NPS response), we calculated the dot-product of a vectorized activation image (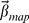) with the NPS pattern (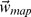), i.e., 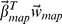, yielding a continuous scalar value. For mediation and other analyses, we used the NPS response calculated from the single-trial beta images.

#### Multilevel two-path mediation analysis

The multilevel mediation analyses based on a standard three-variable path (i.e., two-path) model [103] were performed using the Mediation Toolbox (http://wagerlab.colorado.edu/tools) [6],[8]. The mediation analysis tests whether a covariance between two variables (X and Y) can be explained by a third variable (M). A significant mediator is one whose inclusion as an intermediate variable in a path model of the effects of X on Y significantly affects the slope of the X-Y relationship; that is, the difference (*c−c′*) is statistically significant. In the current study, we used stimulus intensity or self-regulation (regulate-up versus regulate-down) for each trial as the “X” variable and pain report for each trial as the “Y” variable. Thus, the X–Y relationship (path *c*) is the linear association between stimulus intensity or regulation and pain report.

More formally, the three-variable mediation test can be basically captured in a system of three equations:
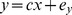


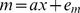


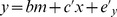
Here *y*, *x*, and *m* are *n* (trials)×1 data vectors for each subject containing the outcome (*y*, the reported pain), the predictor (*x*, stimulus intensity or regulation), and data from a candidate mediating voxel, a cluster, or the NPS response (*m*, activity in single-trial beta images). *e_y_*, *e_m_*, and *e′_y_* vectors denote residual errors for the outcome and mediator controlling for *x* and the outcome controlling for *x* and *m*, respectively. Path *a* is the estimated linear change in *m* per unit change in *x*. Path *b* is the slope of the mediator-outcome relationship controlling for *x*. The paths *c* and *c′* are as described above. Statistical tests on paths *a* and *b* coefficients assess the significance of each relationship. In addition, a statistical test of *(c−c′)* can be performed by testing the significance of the product of the path coefficients of path *a*×*b*.

Based on this first-level mediation model, we conducted multilevel mediation analysis, which is designed for explaining both within- and between-subjects variations in the same model by treating the participant as a random effect (for the details of the method, see Wager and colleagues [8]). This analysis can provide information about brain-behavior relationships at two levels. The first level accounts for the relationships between dynamic variations across time (within individual participants) in stimulus intensity or self-regulation (X), brain activity (M), and pain report (Y). The second level tests for consistency across individuals, allowing population inference, and accounts for known sources of variations in individual pathway strength (i.e., person-level moderators) [104]. Whole brain multilevel mediation analysis tests the mediation effect at each voxel (for more details, see refs. [8],[20]).

We used bootstrapping for significance testing. Bootstrap tests [105] have been shown to be a useful way to assess mediation in small samples [105],. Bootstrapping provides a more accurate and generally more sensitive test for assessing the magnitude of indirect (path *a*×*b*) effects than the Sobel test [107], which assumes a normal distribution of path *a*×*b* estimates. Even if paths *a* and *b* estimates may both be normally distributed, the path *a*×*b* product is not expected to be normally distributed [108]. We estimated distributions of subject-level path coefficients by randomly sampling with replacement 10,000 observations (rows) from the matrix of [*a b c′ c* (*a*×*b*)] path coefficients. Two-tailed *p*-values were calculated from the bootstrap confidence interval.

In order to test whether the NPS response mediated the relationship between self-regulation and pain report, we conducted two multilevel mediation analyses (Figure 3). As explained above, X was self-regulation (regulate-up versus regulate-down) (Figure 3A) or stimulus intensity (Figure 3B), Y was pain ratings, and M was the NPS responses that were calculated from single-trial beta images. In the mediation model with stimulus intensity as X, self-regulation was included as a covariate, and in the model with self-regulation as X, stimulus intensity was included as a covariate. In addition, we conducted two whole-brain searches with multilevel mediation analysis to identify brain mediators of the effects of stimulus intensity and self-regulation on pain (Figure S6). Covariates were included in the same way as above.

#### Multilevel three-path mediation analysis

The three-path mediation analysis can assess relationships among stimulus intensity or self-regulation (X), two different brain mediators (M1 and M2), and pain report (Y). The analysis is based on a three-path mediation model suggested by [109].

Adopting the notational convention of [109], the three-path mediation model can be captured in a system of the following four equations and a diagram (Figure S7):
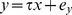


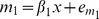


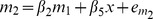


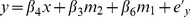
Here, we are interested in the effects mediated by both mediators (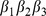). We used two different criteria of testing for the three-path mediation effects, and only if variables met both criteria, they were considered to be significant mediators: (1) the joint significance test: Each of the three paths (i.e., 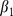, 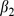, 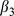) should be significant [108], and (2) the product-of-coefficients test using bootstrap test: The product of coefficients, 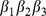 (its sample estimate is *b*_1_ *b*_2_ *b*_3_), should be significantly nonzero. These two criteria were shown to be better than other methods in terms of type I and type II errors [109].

The multilevel implementation is same with the two-path mediation analysis (for the details, see [8]) except for the calculation of the variance of the mediated path (*b*_1_ *b*_2_ *b*_3_). In the two-path mediation analysis, we used the following equation from [104]:
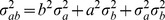
Here, *a* and *b* is path estimate for path a and b. In the three-path mediation analysis, we used the multivariate delta estimator using the following equation from [109]:
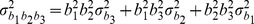
This variance estimate was used in Empirical Bayes estimation procedure for second-level bootstrapping of the path coefficients [8].

To identify potential pathways connecting self-regulation and reported pain, we used two *a priori* ROIs (the NAc and vmPFC) as the first and second mediators (Figure 5). The ROI coordinates were from [53]. For the ROI values, we averaged activity across voxels within a sphere (*r* = 6 mm) around the ROI centers. In order to control for nociception-related brain activity, stimulus intensity and the NPS response were included as covariates.

In addition to the ROI analysis, we conducted whole-brain searches using multilevel three-path mediation analysis, where three variables (self-regulation, pain reports, and one brain region) were specified *a priori*, and a voxel-wise search was conducted for mediators. For the first whole-brain search (Figure 6A), left NAc (MNI, −14, 8, −8) from the robust regression results for the regulate-up versus regulate-down contrast (Figure 4A) was used as the first mediator (M1). For the second whole-brain search (Figure 6B), vmPFC (MNI, 2, 52, −2) from the first whole-brain search was used as the second mediator (M2). Consistent with the ROI analysis, stimulus intensity and the NPS response were included as covariates. Matlab codes implementing all analyses is available at http://wagerlab.colorado.edu/tools.

## Supporting Information

Figure S1 **Experimental design.** The experiment consisted of nine runs. Among the runs, the third and seventh were “regulation” runs, which consisted of “regulate-up (increase pain)” and “regulate-down (decrease pain)”. The order of the two conditions was counterbalanced across subjects. Passive experience (i.e., no regulation) runs comprised 11 trials, and regulation runs comprised ten trials. During each run, thermal stimulations that consisted of five levels of intensity were delivered. The regulate-up or -down instructions were presented before regulation runs (third or seventh runs). Every run started with a baseline period during which a fixation cross was presented for 18 seconds. Each trial started with a 12.5-second long thermal stimulation, followed by a 4.5- to 8.5- second long pre-rating period. After the pre-rating period, participants were asked to decide if the stimulation was painful or not. Then, participants rated the intensity of the warmth or painful sensation on a scale of 0 to 100. A 5- to 9-second inter-trial interval followed the rating period.
(TIF)

Figure S2 **The effects of stimulus intensity and self-regulation on pain/no-pain decisions.** (A) The percentage of trials on which people reported the stimulus was painful, as a function of stimulus intensity and regulation conditions. (B) The main effects of manipulations (stimulus intensity and regulate-up versus regulate-down) on the percentage of pain decision. Beta (y-axis) represents regression coefficients from logistic regression for each participant, and error bars represent standard errors of the mean (SEM) across participants. **p<0.01; ***p<0.001, two-tailed.
(TIF)

Figure S3 **Stimulus intensity-induced brain activity.** All colored regions were significant at *p*<0.05, FWER corrected based on cluster extent. The legend indicates primary voxel-wise threshold levels and cluster extent threshold (parentheses). For the purpose of display, we pruned the results using two additional higher levels of voxel-wise threshold.
(TIF)

Figure S4 **The effects of manipulations on brain regions associated with self-regulation.** (A) Activity in caudate (left) and sensory motor cortex (SMC) were associated with regulate-up versus -down instructions (at *p*<0.05, FWER corrected based on cluster extent, with a primary threshold of *p*<0.0005). (B) Superior parietal lobe (right) was associated with regulation versus passive experience instructions. (C) Bar plots of the averaged activity (y-axis) across voxels within the corresponding brain region for regulation conditions (x-axis). Error bars represent within-subject standard errors of the mean (SEM).
(TIF)

Figure S5 **Brain activity for regulate-up and regulate-down.** (A) Brain regions that are associated with regulate-up versus passive experience. (B) Brain regions that are associated with regulate-down versus passive experience. All colored regions were significant at *p*<0.05, FWER corrected based on cluster extent estimated by Monte-Carlo simulation. The legend indicates primary voxel-wise threshold levels and cluster extent threshold (parentheses).
(TIF)

Figure S6 **Whole-brain search for mediators of the relationship between stimulus intensity/self-regulation and pain rating.** (A) Left: A mediation model for the effects on stimulus intensity on pain. Right: The results of the mediation analysis. (B) Left: A mediation model for the self-regulation effects on pain. Right: The mediation analysis results. Here, we show path *a*, *b*, and their conjunction. Path *a*: Significant brain regions in the path *a* of the mediation model. In path *a*, self-regulation (regulate-up versus -down) was the predictor, and brain voxel activity was the outcome. Path *b*: significant brain regions in the path *b*, where brain voxel activity was the predictor, and pain report was the outcome. Conjunction: conjunction maps between (a) *positive* regions in path *a* and path *b* and between (b) *negative* regions in path *a* and path *b*. Yellow and cyan colors show the overlapped regions between path *a* and path *b*. There was only one small cluster that showed overlaps between path *a* and path *b*, but the cluster did not survive correction for multiple comparisons. All maps were thresholded at cluster-extent based threshold *p*<0.05, FWER corrected based on Monte-Carlo simulation. The legend indicates primary threshold levels and cluster extent sizes (in parentheses).
(TIF)

Figure S7 **Path diagram of the three-path mediation model.** This diagram is modified from Taylor and colleagues [109].
(TIF)

Table S1 **Pain ratings and neurologic pain signature response for each condition.** Numerical data to draw Figure 1C and 1D: mean and standard errors of pain ratings and NPS responses for experimental conditions (stimulus intensity and self-regulation). Within-subject standard errors of the mean (SEM) are shown in parenthesis.
(TIF)

Table S2 **Effects of manipulations on the neurologic pain signature sub-region response.** Numerical data to draw a bar plot in Figure 2B: The main effects of stimulus intensity and self-regulation on the NPS response within sub-regions. *β* represents standardized regression coefficients from a multilevel generalized linear model with stimulus intensity and self-regulation (regulate-up versus -down) as predictors and NPS response as dependent variables. SEM represents standard errors of the mean.
(TIF)

Table S3 **Stimulus intensity-related brain activity.** The reported regions were significantly associated with by stimulus intensity (parametric modulation). The results were significant at *p*<0.05, FWER corrected based on cluster extent (*k*>3), with a primary threshold of *p*<0.00001. The size of cluster extent for FWER correction was estimated based on Monte Carlo simulation. AG, angula gyrus; FP, frontal pole; INS, insula; LG, lingual gyrus; MFG, middle frontal gyrus; MPFC, medial prefrontal cortex; MTG, middle temporal gyrus; OG, occipital gyrus; PCC, posterior cingulate cortex; PAG, peryaqueductal gray; PHG, parahippocampal gyrus; PCC, posterior cingulate cortex; SMA, supplementary motor area; SMG, supramarginal gyrus; STG, superior temporal gyrus.
(TIF)

Table S4 **Self-regulation-induced brain activity.** The reported regions were significantly associated with regulate-up versus regulate-down and regulation versus passive experience instructions (parametric modulation). The results were significant at *p*<0.05, FWER corrected based on cluster extent (*k*>84), with a primary threshold of *p*<0.0005. The size of cluster extent for FWER correction was estimated based on Monte Carlo simulation. Activation values are numerical data to draw a bar plot in Figure 4C: The activation values represent the averaged activity within the region for each experimental condition. Within-subject standard errors of the mean (SEM) are shown in parenthesis. IFJ, inferior frontal junction; SMA, supplementary motor area; SPL, superior parietal lobe.
(TIF)

Table S5 **Path coefficients for whole-brain three-path mediation analyses.** The results of two whole-brain three-path mediation analyses are presented. In both mediation models, X was a regressor for the contrast of regulate-up (1) versus passive experience (0) versus regulate-down (−1). (A) In the first mediation model, the left NAc (MNI coordinate: −14, 8, −8), which was a brain region significantly associated with regulate-up versus -down instructions (Figure 4A), was entered as the first mediator (M1), and we searched for significant second mediators (M2) of the relationship between the regulation (X)-NAc (M1) connection and pain rating (Y) in the whole-brain. The result showed the vmPFC (mm center = 2, 52, −2) was the only significant second mediator. (B) In the second mediation model, vmPFC that was the significant second mediator from the first whole-brain three-path mediation analysis was entered as the second mediator (M2), and we searched for significant first mediators (M1) of the relationship between regulation (X) and the vmPFC (M2)-Pain rating (Y) connection. The result showed right NAc (mm center = 8, 8, −6) was the only significant first mediator. All results were thresholded at *p*<0.05, FWER corrected based on cluster extent, with a primary threshold of *p*<0.001. The size of cluster extent for FWER correction was estimated based on Monte Carlo simulation (*k*>17 and 18 for A and B, respectively). M1, the first mediator; M2, the second mediator.
(TIF)

## Abbreviations

aINS: anterior insula
dACC: dorsal anterior cingulate cortex
dpINS: dorsal-posterior insula
fMRI: functional magnetic resonance imaging
FWER: family-wise error rate
NAc: nucleus accumbens
NPS: neurologic pain signature
ROI: region-of-interest
S2: secondary somatosensory cortices
vmPFC: ventromedial prefrontal cortex
