# Supplementary figures and images for: Distinct Brain Systems Mediate the Effects of Nociceptive Input and Self-Regulation on Pain
Source: PLoS Biol. 2015 Jan 6;13(1):e1002036. doi: 10.1371/journal.pbio.1002036 (PMC4285399; doi:10.1371/journal.pbio.1002036)

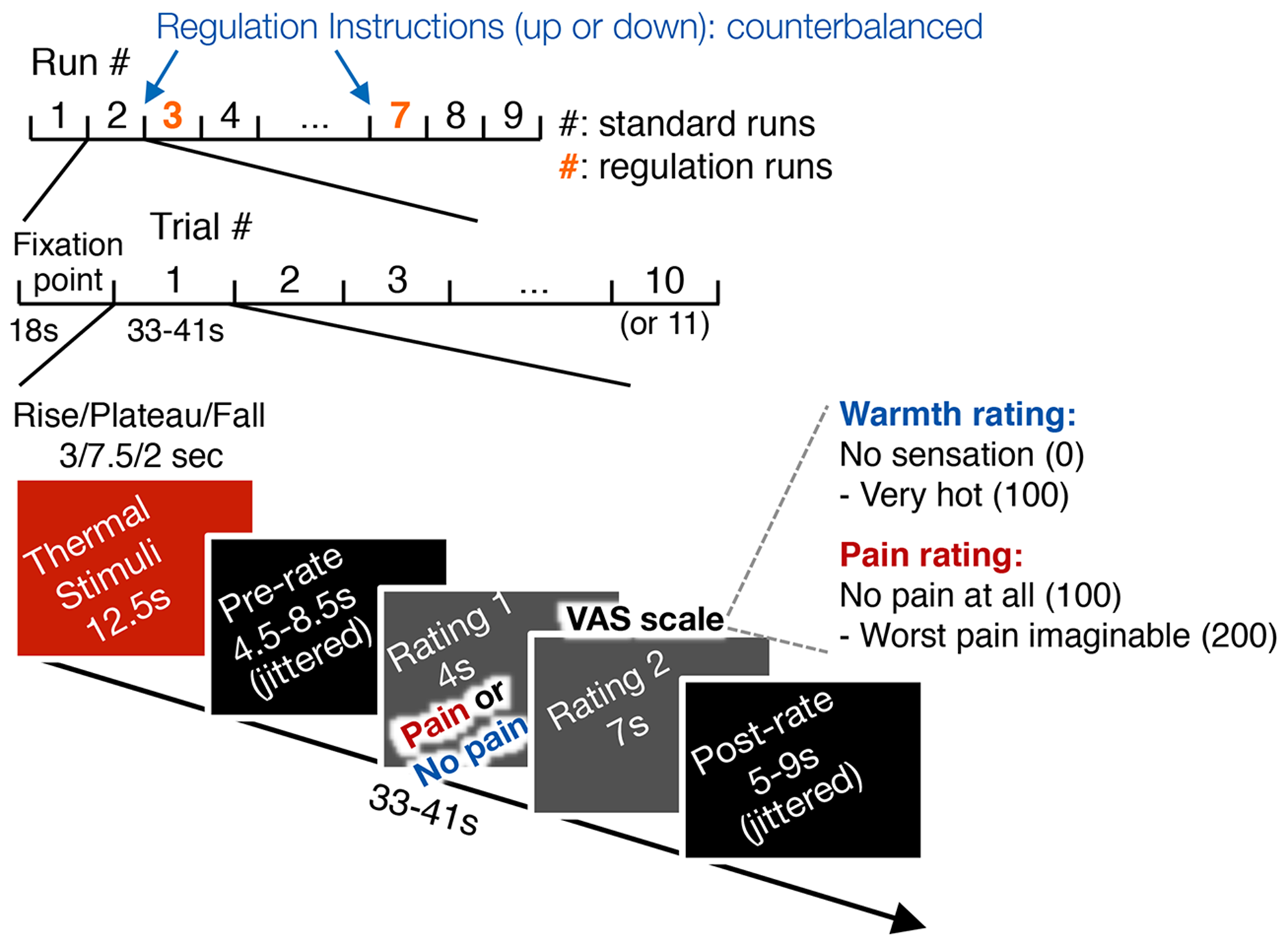

Supplement: Figure S1 — Experimental design. The experiment consisted of nine runs. Among the runs, the third and seventh were “regulation” runs, which consisted of “regulate-up (increase pain)” and “regulate-down (decrease pain)”. The order of the two conditions was counterbalanced across subjects. Passive experience (i.e., no regulation) runs comprised 11 trials, and regulation runs comprised ten trials. During each run, thermal stimulations that consisted of five levels of intensity were delivered. The regulate-up or -down instructions were presented before regulation runs (third or seventh runs). Every run started with a baseline period during which a fixation cross was presented for 18 seconds. Each trial started with a 12.5-second long thermal stimulation, followed by a 4.5- to 8.5- second long pre-rating period. After the pre-rating period, participants were asked to decide if the stimulation was painful or not. Then, participants rated the intensity of the warmth or painful sensation on a scale of 0 to 100. A 5- to 9-second inter-trial interval followed the rating period. (TIF) [file pbio.1002036.s001.tif]

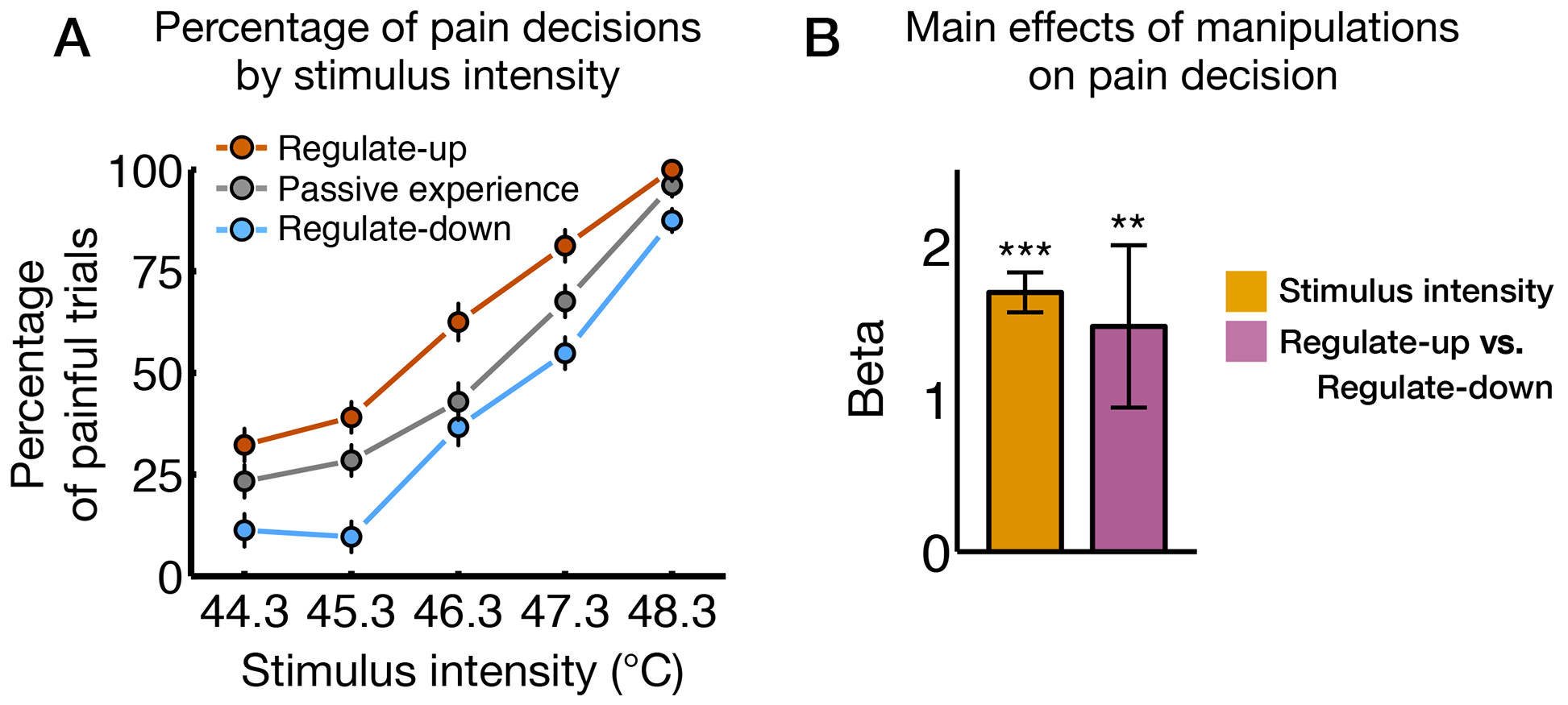

Supplement: Figure S2 — The effects of stimulus intensity and self-regulation on pain/no-pain decisions. (A) The percentage of trials on which people reported the stimulus was painful, as a function of stimulus intensity and regulation conditions. (B) The main effects of manipulations (stimulus intensity and regulate-up versus regulate-down) on the percentage of pain decision. Beta (y-axis) represents regression coefficients from logistic regression for each participant, and error bars represent standard errors of the mean (SEM) across participants. **p<0.01; ***p<0.001, two-tailed. (TIF) [file pbio.1002036.s002.tif]

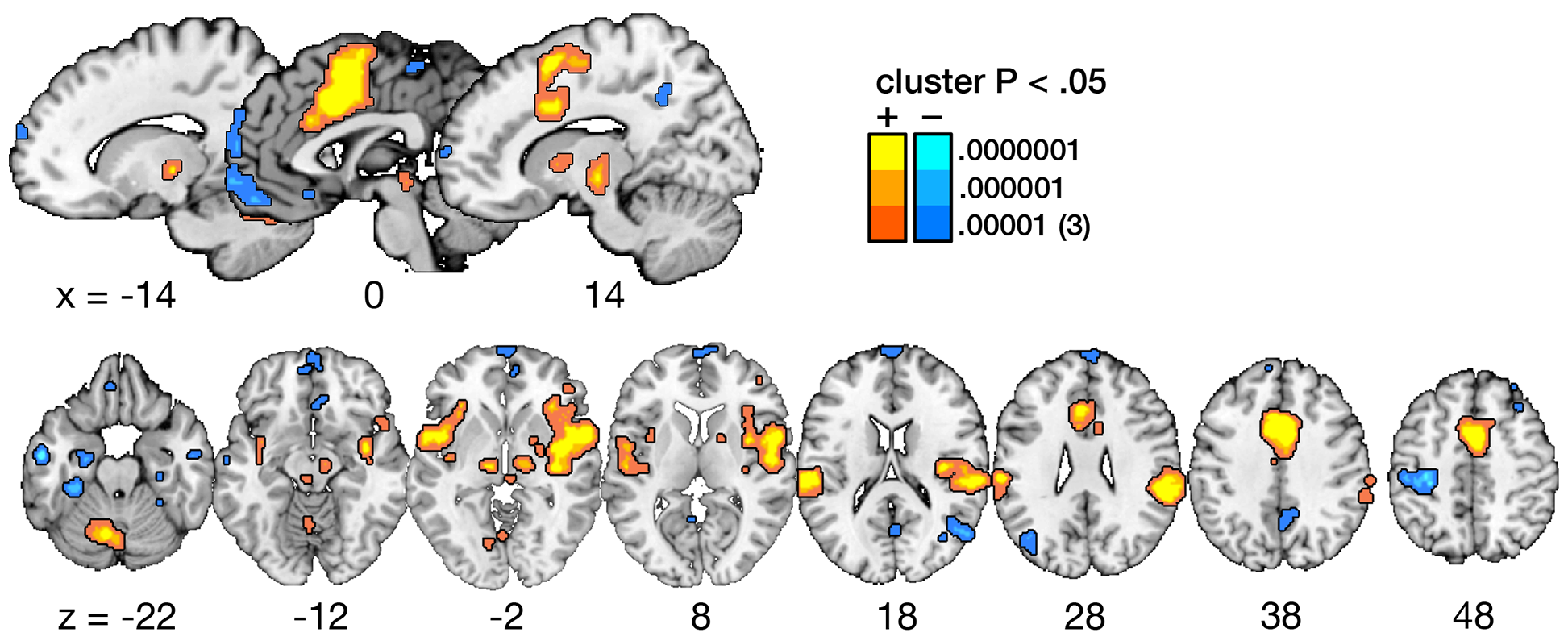

Supplement: Figure S3 — Stimulus intensity-induced brain activity. All colored regions were significant at p<0.05, FWER corrected based on cluster extent. The legend indicates primary voxel-wise threshold levels and cluster extent threshold (parentheses). For the purpose of display, we pruned the results using two additional higher levels of voxel-wise threshold. (TIF) [file pbio.1002036.s003.tif]

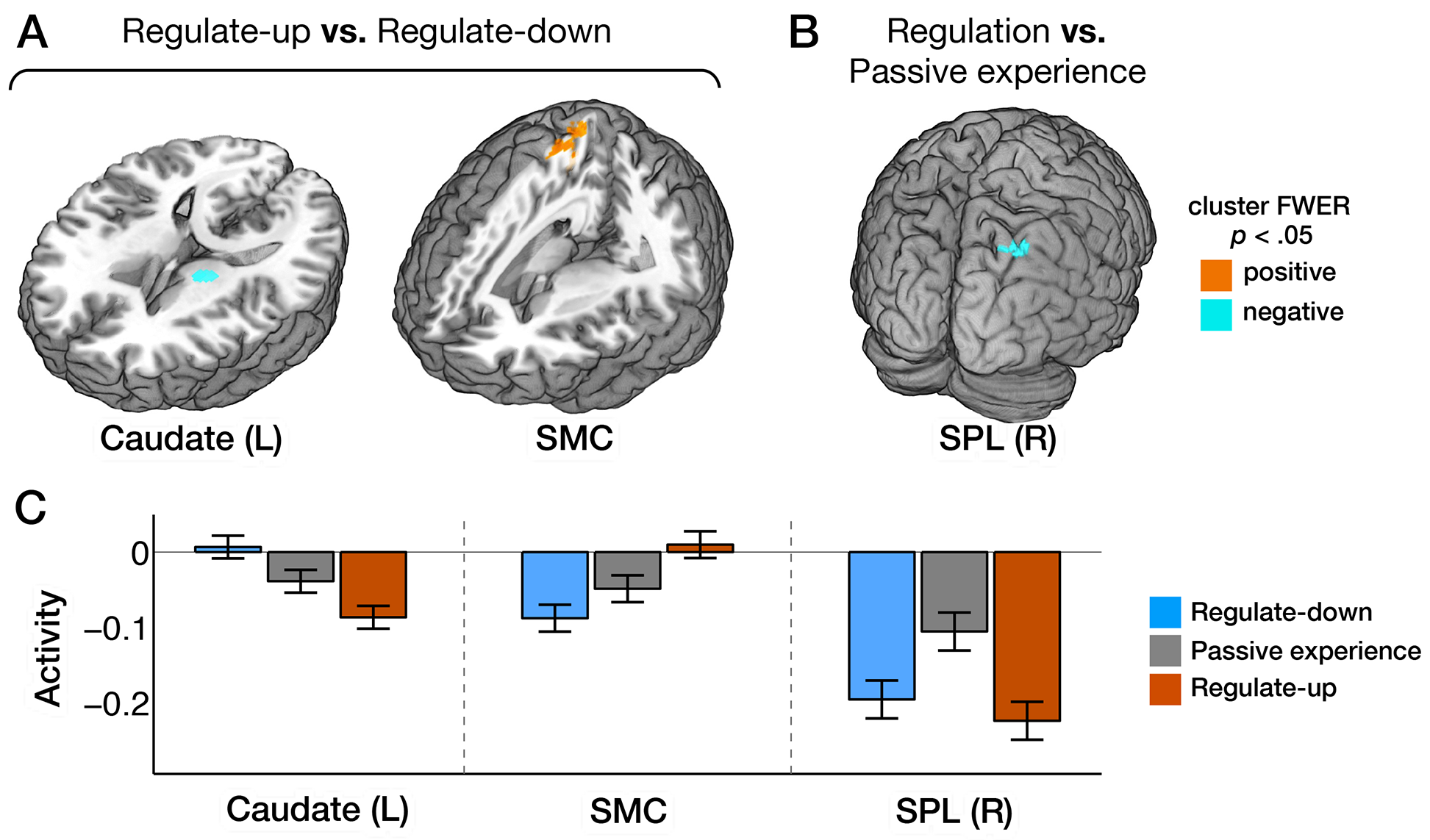

Supplement: Figure S4 — The effects of manipulations on brain regions associated with self-regulation. (A) Activity in caudate (left) and sensory motor cortex (SMC) were associated with regulate-up versus -down instructions (at p<0.05, FWER corrected based on cluster extent, with a primary threshold of p<0.0005). (B) Superior parietal lobe (right) was associated with regulation versus passive experience instructions. (C) Bar plots of the averaged activity (y-axis) across voxels within the corresponding brain region for regulation conditions (x-axis). Error bars represent within-subject standard errors of the mean (SEM). (TIF) [file pbio.1002036.s004.tif]

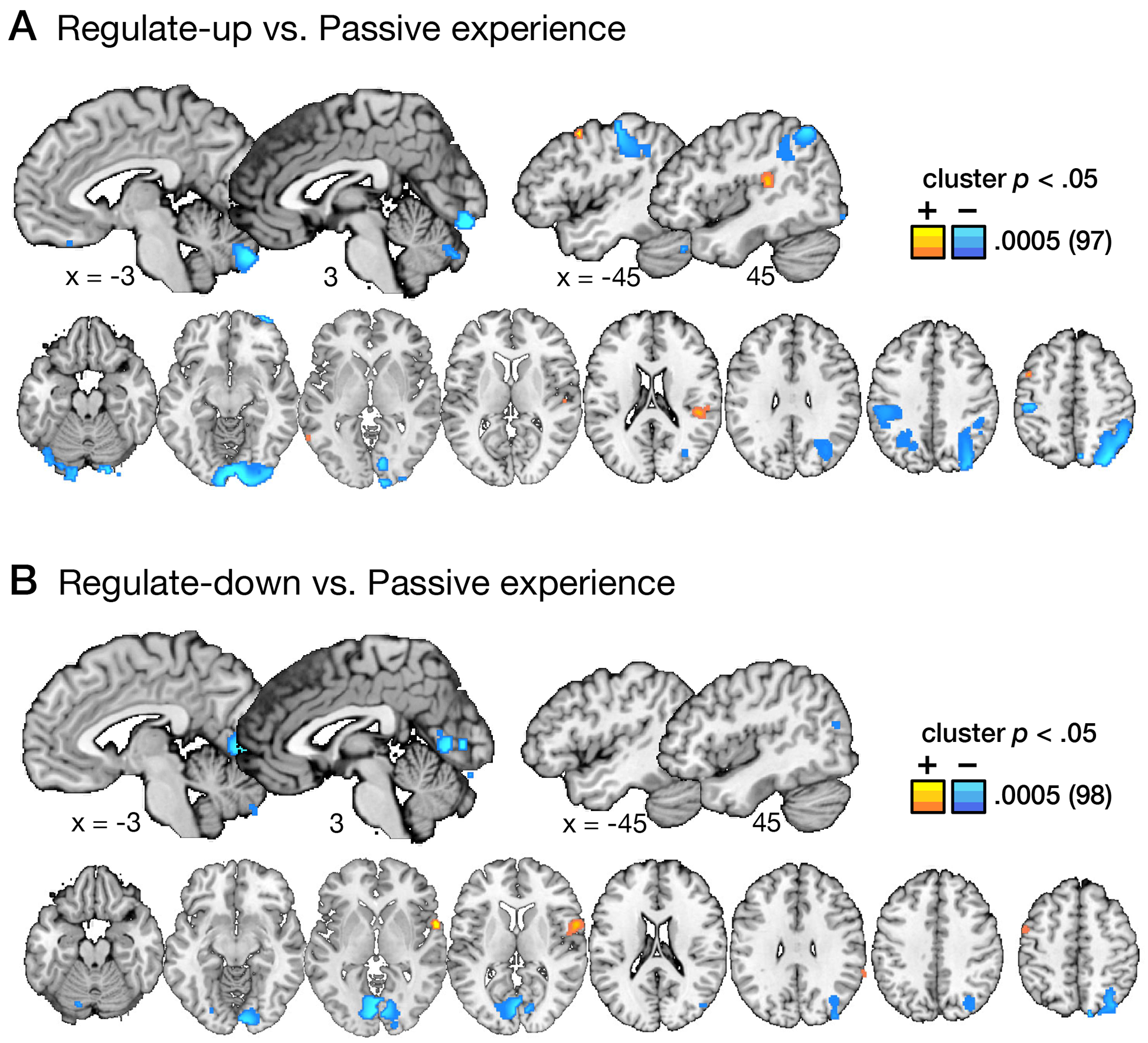

Supplement: Figure S5 — Brain activity for regulate-up and regulate-down. (A) Brain regions that are associated with regulate-up versus passive experience. (B) Brain regions that are associated with regulate-down versus passive experience. All colored regions were significant at p<0.05, FWER corrected based on cluster extent estimated by Monte-Carlo simulation. The legend indicates primary voxel-wise threshold levels and cluster extent threshold (parentheses). (TIF) [file pbio.1002036.s005.tif]

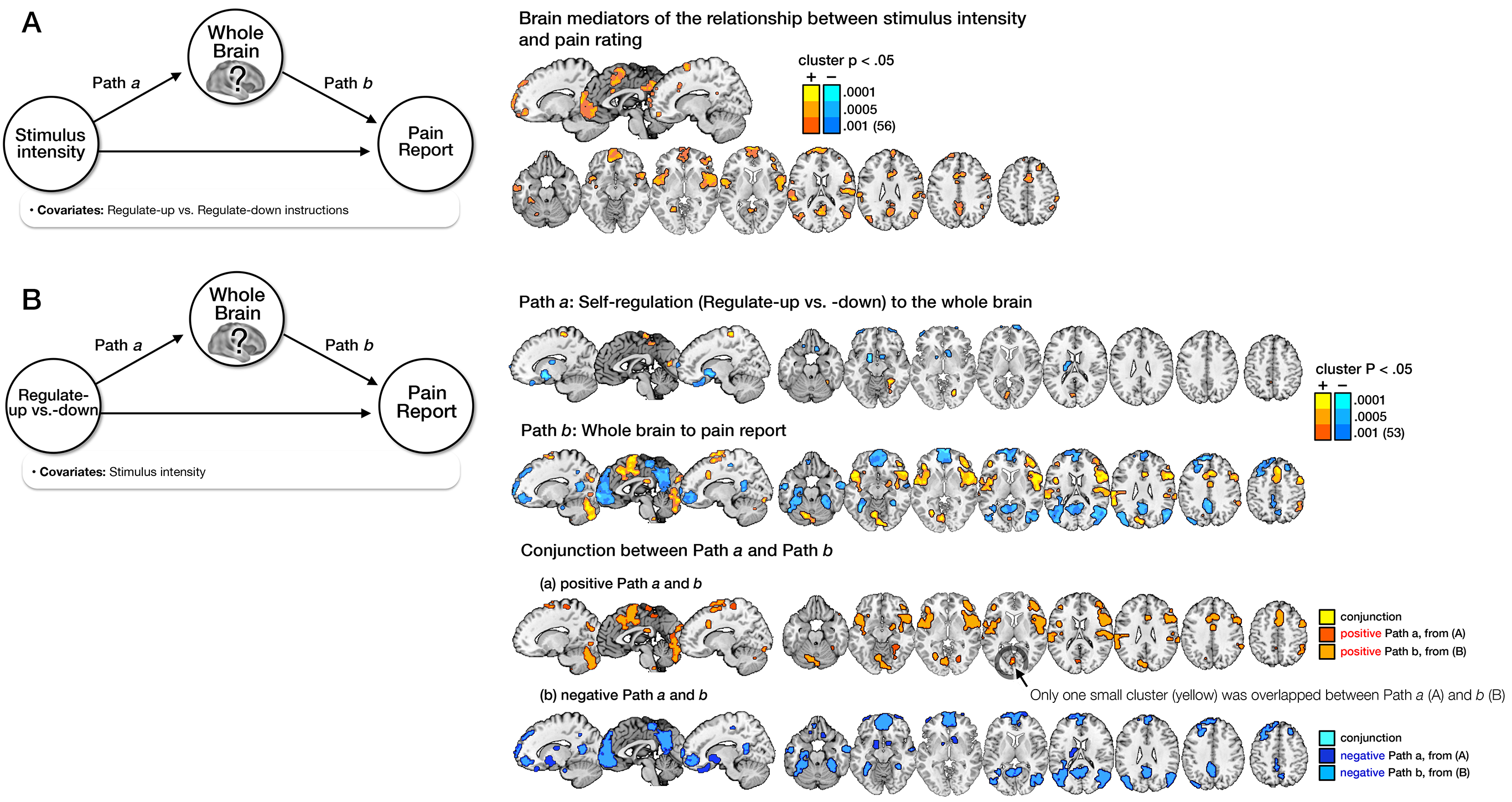

Supplement: Figure S6 — Whole-brain search for mediators of the relationship between stimulus intensity/self-regulation and pain rating. (A) Left: A mediation model for the effects on stimulus intensity on pain. Right: The results of the mediation analysis. (B) Left: A mediation model for the self-regulation effects on pain. Right: The mediation analysis results. Here, we show path a, b, and their conjunction. Path a: Significant brain regions in the path a of the mediation model. In path a, self-regulation (regulate-up versus -down) was the predictor, and brain voxel activity was the outcome. Path b: significant brain regions in the path b, where brain voxel activity was the predictor, and pain report was the outcome. Conjunction: conjunction maps between (a) positive regions in path a and path b and between (b) negative regions in path a and path b. Yellow and cyan colors show the overlapped regions between path a and path b. There was only one small cluster that showed overlaps between path a and path b, but the cluster did not survive correction for multiple comparisons. All maps were thresholded at cluster-extent based threshold p<0.05, FWER corrected based on Monte-Carlo simulation. The legend indicates primary threshold levels and cluster extent sizes (in parentheses). (TIF) [file pbio.1002036.s006.tif]

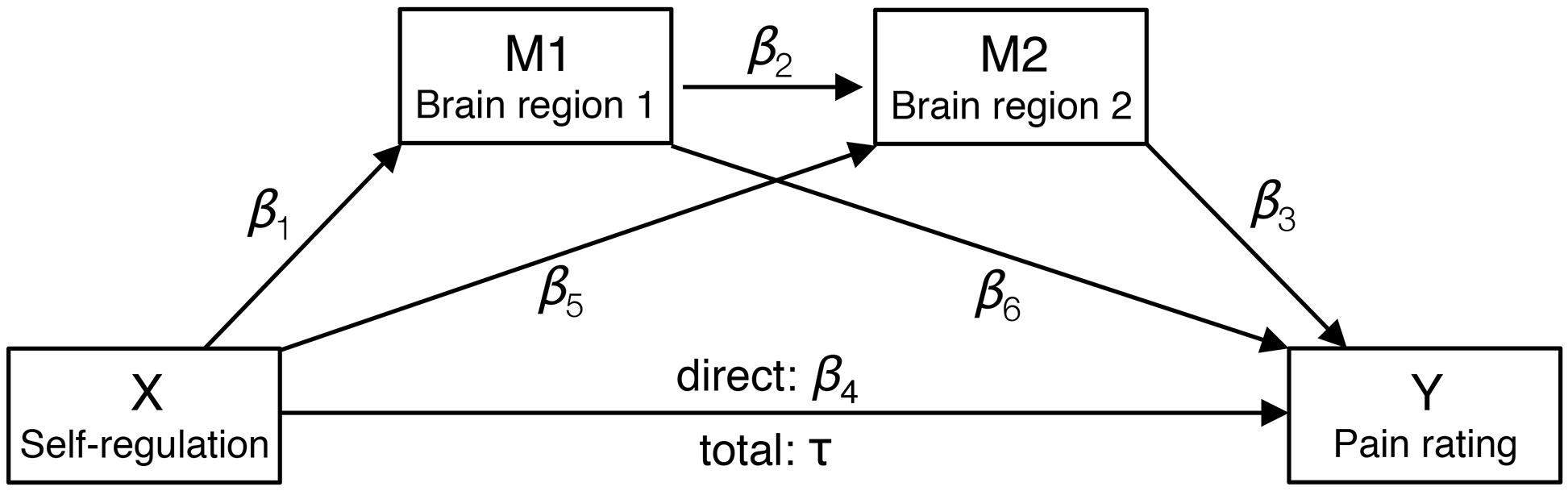

Supplement: Figure S7 — Path diagram of the three-path mediation model. This diagram is modified from Taylor and colleagues [109]. (TIF) [file pbio.1002036.s007.tif]

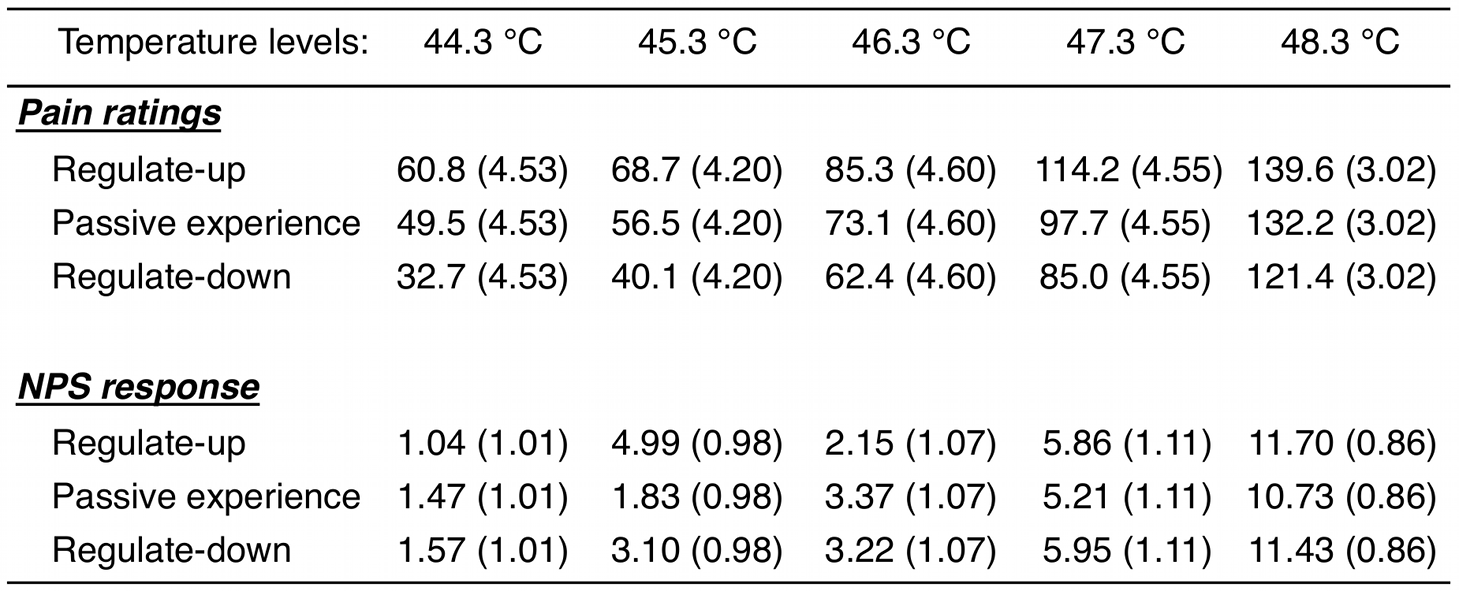

Supplement: Table S1 — Pain ratings and neurologic pain signature response for each condition. Numerical data to draw Figure 1C and 1D: mean and standard errors of pain ratings and NPS responses for experimental conditions (stimulus intensity and self-regulation). Within-subject standard errors of the mean (SEM) are shown in parenthesis. (TIF) [file pbio.1002036.s008.tif]

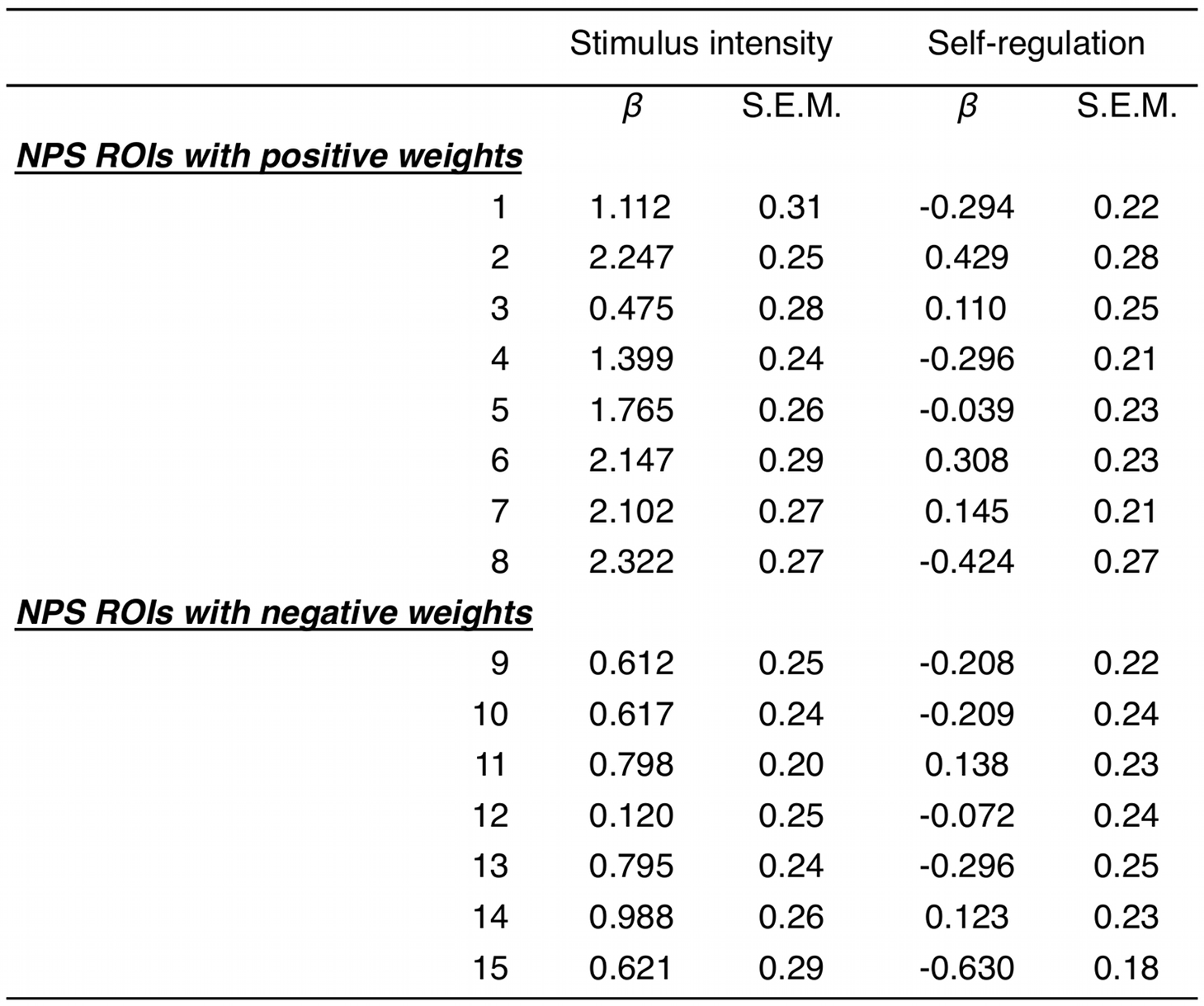

Supplement: Table S2 — Effects of manipulations on the neurologic pain signature sub-region response. Numerical data to draw a bar plot in Figure 2B: The main effects of stimulus intensity and self-regulation on the NPS response within sub-regions. β represents standardized regression coefficients from a multilevel generalized linear model with stimulus intensity and self-regulation (regulate-up versus -down) as predictors and NPS response as dependent variables. SEM represents standard errors of the mean. (TIF) [file pbio.1002036.s009.tif]

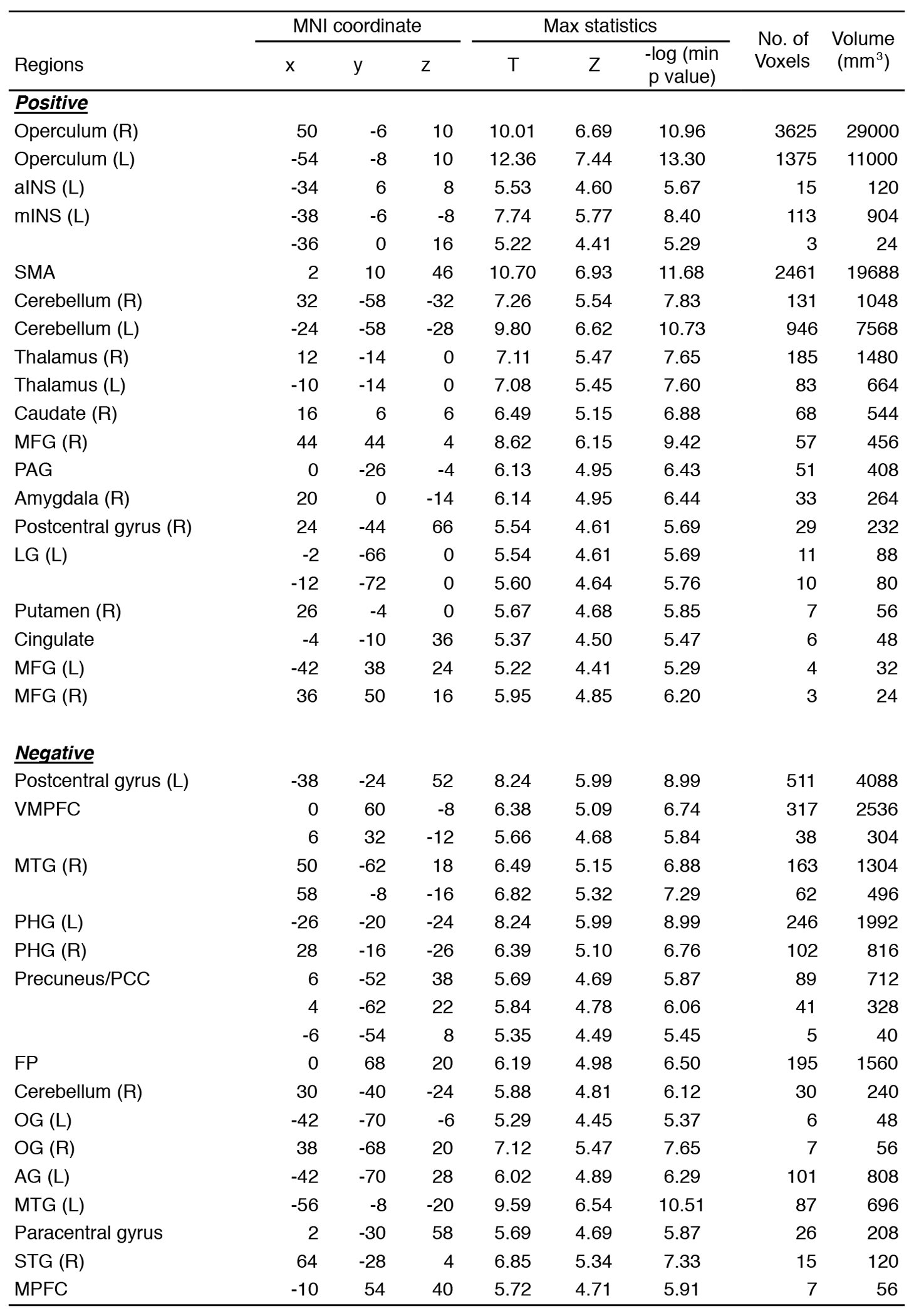

Supplement: Table S3 — Stimulus intensity-related brain activity. The reported regions were significantly associated with by stimulus intensity (parametric modulation). The results were significant at p<0.05, FWER corrected based on cluster extent (k>3), with a primary threshold of p<0.00001. The size of cluster extent for FWER correction was estimated based on Monte Carlo simulation. AG, angula gyrus; FP, frontal pole; INS, insula; LG, lingual gyrus; MFG, middle frontal gyrus; MPFC, medial prefrontal cortex; MTG, middle temporal gyrus; OG, occipital gyrus; PCC, posterior cingulate cortex; PAG, peryaqueductal gray; PHG, parahippocampal gyrus; PCC, posterior cingulate cortex; SMA, supplementary motor area; SMG, supramarginal gyrus; STG, superior temporal gyrus. (TIF) [file pbio.1002036.s010.tif]

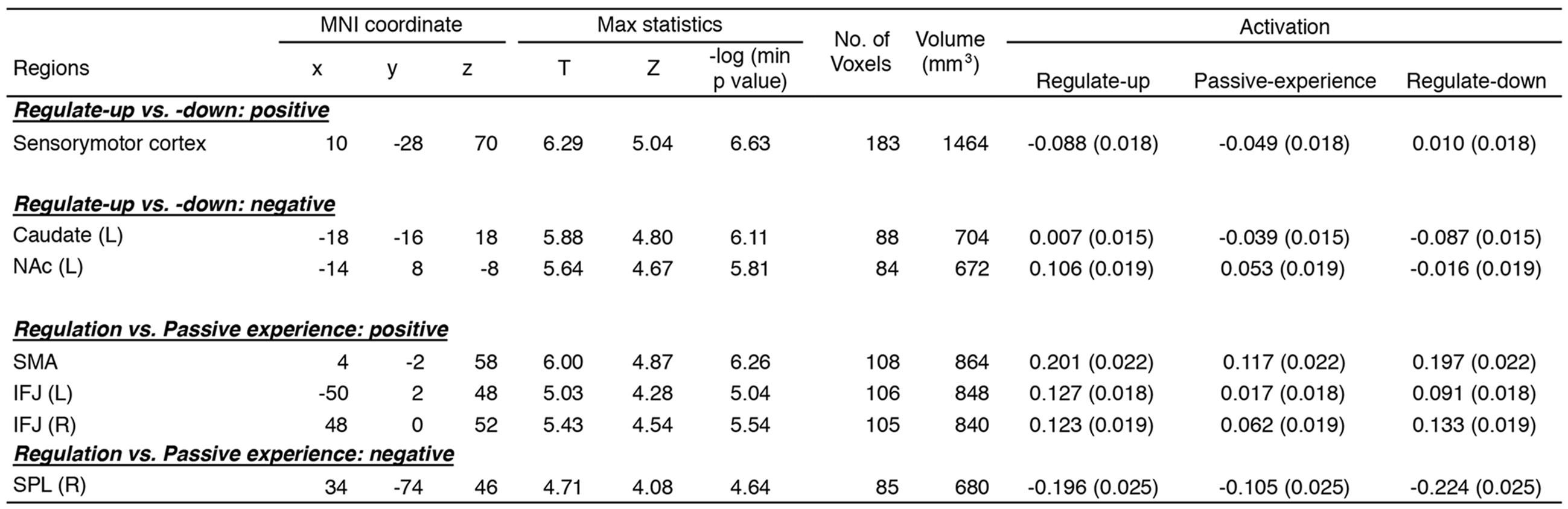

Supplement: Table S4 — Self-regulation-induced brain activity. The reported regions were significantly associated with regulate-up versus regulate-down and regulation versus passive experience instructions (parametric modulation). The results were significant at p<0.05, FWER corrected based on cluster extent (k>84), with a primary threshold of p<0.0005. The size of cluster extent for FWER correction was estimated based on Monte Carlo simulation. Activation values are numerical data to draw a bar plot in Figure 4C: The activation values represent the averaged activity within the region for each experimental condition. Within-subject standard errors of the mean (SEM) are shown in parenthesis. IFJ, inferior frontal junction; SMA, supplementary motor area; SPL, superior parietal lobe. (TIF) [file pbio.1002036.s011.tif]

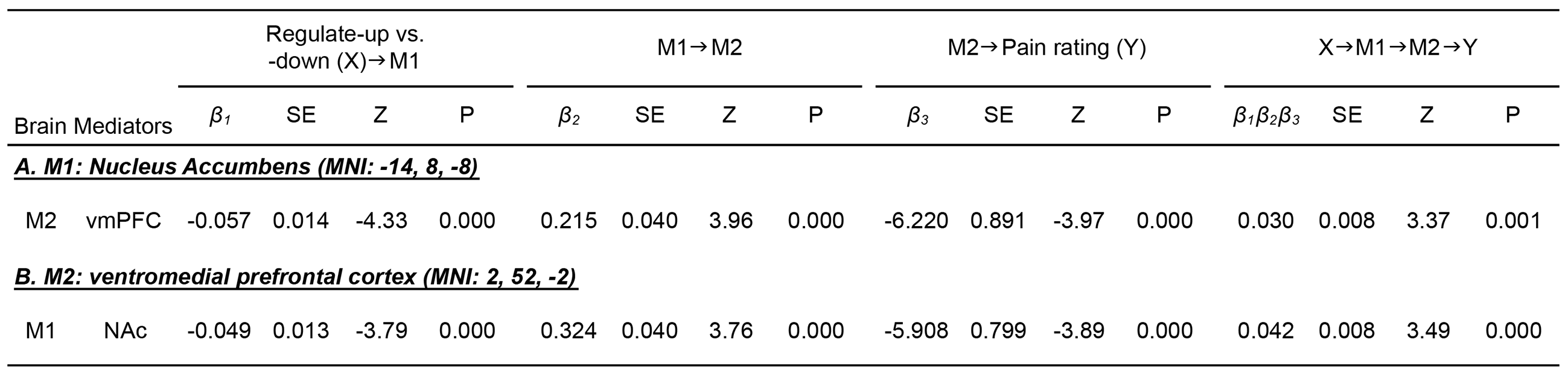

Supplement: Table S5 — Path coefficients for whole-brain three-path mediation analyses. The results of two whole-brain three-path mediation analyses are presented. In both mediation models, X was a regressor for the contrast of regulate-up (1) versus passive experience (0) versus regulate-down (−1). (A) In the first mediation model, the left NAc (MNI coordinate: −14, 8, −8), which was a brain region significantly associated with regulate-up versus -down instructions (Figure 4A), was entered as the first mediator (M1), and we searched for significant second mediators (M2) of the relationship between the regulation (X)-NAc (M1) connection and pain rating (Y) in the whole-brain. The result showed the vmPFC (mm center = 2, 52, −2) was the only significant second mediator. (B) In the second mediation model, vmPFC that was the significant second mediator from the first whole-brain three-path mediation analysis was entered as the second mediator (M2), and we searched for significant first mediators (M1) of the relationship between regulation (X) and the vmPFC (M2)-Pain rating (Y) connection. The result showed right NAc (mm center = 8, 8, −6) was the only significant first mediator. All results were thresholded at p<0.05, FWER corrected based on cluster extent, with a primary threshold of p<0.001. The size of cluster extent for FWER correction was estimated based on Monte Carlo simulation (k>17 and 18 for A and B, respectively). M1, the first mediator; M2, the second mediator. (TIF) [file pbio.1002036.s012.tif]
